# Advances in GLP-1 receptor agonists delivery systems for obesity and diabetes

**DOI:** 10.1016/j.apsb.2026.01.035

**Published:** 2026-01-31

**Authors:** Mehrnaz Abbasi, Kai Sun, Kevin W. Huggins, Braeden Heath, Hannah DeLoit, Lauren McGinness, Kate Mccamy

**Affiliations:** a Department of Nutritional Sciences, College of Human Sciences, Auburn University, Auburn, AL 36849, USA; b Center for Metabolic and Degenerative Diseases, The Brown Foundation Institute of Molecular Medicine for the Prevention of Human Diseases, University of Texas Health Science Center at Houston, Houston, TX, USA; Graduate School of Biomedical Sciences (GSBS), University of Texas Health Science Center at Houston, Houston, TX 77030, USA; c Department of Biomedical Sciences, College of Sciences and Mathematics, Auburn University, Auburn, AL 36849, USA; d Department of Pre-Health Professional Curricula, College of Sciences and Mathematics, Auburn University, Auburn, AL 36849, USA

**Keywords:** Glucagon-like peptide-1 receptor agonists, GLP-1, Advanced delivery systems, Nanoparticle, Microneedle, Nano/microcarrier-based delivery systems, Obesity, Diabetes

## Abstract

Obesity and diabetes are chronic metabolic diseases affecting millions worldwide. Current treatments, including lifestyle changes, medications, and surgery, face challenges like poor adherence and side effects. Glucagon-like peptide-1 receptor agonists (GLP-1RAs) are recommended innovative medications for these conditions, with studies showing significant clinical benefits. GLP-1RAs are traditionally delivered orally or *via* subcutaneous injections, but these methods have limitations, including low bioavailability, poor solubility, the need for high doses, gastrointestinal side effects, and frequent dosing requirements. Novel delivery technologies offer promising strategies to overcome these challenges and enhance therapeutic effectiveness. Recent advances in drug delivery technologies, including nanocarrier- and microcarrier-based systems, hydrogels, microneedles, and innovative formulations such as long-acting, co- and/or nano-formulated agents, offer promising strategies to enhance the delivery, efficacy, and patient adherence of GLP-1RAs for obesity and diabetes. This review focuses on innovative delivery technologies developed for three main GLP-1RAs: exenatide, liraglutide, and semaglutide. We present a review of advancements in drug delivery systems, exploring technologies employed in the development of these agents, as well as future challenges. It is crucial to note that these technologies are still in early development, and further studies are needed to confirm their long-term safety, efficacy, and cost-effectiveness in clinical use.

## Graphical abstract

Obesity and diabetes, which affect millions, could benefit from innovative GLP-1R agonists and advanced delivery technologies. These advancements may overcome current treatment limitations, improving effectiveness and patient adherence.

**Figure:**
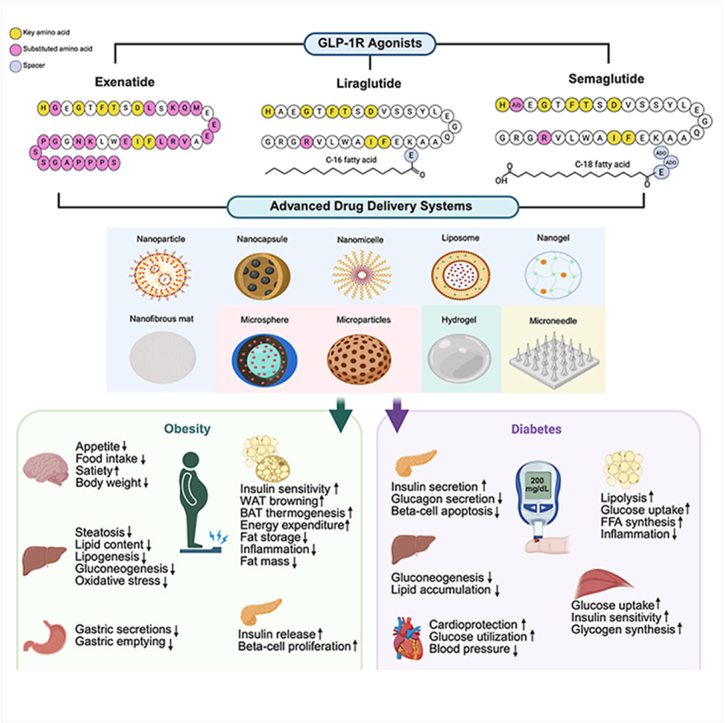

## 1 Introduction

### 1.1 Obesity

Obesity is characterized by excessive accumulation or abnormal distribution of adipose tissue, which presents serious health risks and requires long-term management strategies^1^^,^^2^. Its global prevalence has dramatically increased in recent decades, with current estimates indicating that over one billion individuals are affected worldwide^3^. Obesity typically results from an energy imbalance where caloric intake exceeds energy expenditure, leading to weight gain and increased body fat^4^. However, it is a multifactorial and complex disorder that results from an interplay of genetic, cultural, and societal factors. Other contributors to obesity include sedentary lifestyle, disrupted sleep patterns, hormonal imbalances, certain medications (*e.g.*, insulin, sulfonylureas, *β*-blockers, antidepressants like amitriptyline, corticosteroids like prednisone, etc.), high-calorie and sugary fast foods, and reduced metabolic rates^5^^,^^6^.

Obesity adversely impacts nearly all body systems and is strongly associated with severe chronic diseases such as diabetes, heart disease, and cancer^1^^,^^7^. While it has been suggested that effective obesity treatment combines lifestyle modification and medication^8^, maintaining these lifestyle modifications long-term is challenging and typically results in only 5%–6% weight loss in six months^9^^,^^10^. Current FDA-approved weight loss medications are short-term sympathomimetics like phentermine and long-term options like liraglutide and semaglutide, which can help achieve weight loss of up to 16%. Metformin is sometimes used off-label, and medication selection depends on individual health conditions^11^, ^12^, ^13^. Bariatric surgery is considered for those with a BMI ≥35 kg/m^2^ and related health issues when other interventions fail, but it carries risks that require specialized care^14^^,^^15^. Despite the availability of anti-obesity medicines, many face challenges such as poor bioavailability, rapid clearance, and instability, which refers to the drugs’ susceptibility to chemical, physical, or enzymatic degradation during production, storage, or after administration. Chemical processes like hydrolysis, oxidation, and enzymatic cleavage particularly affect peptide-based drugs such as GLP-1 receptor agonists (GLP-1RAs) (*e.g.*, liraglutide and semaglutide), causing rapid degradation and loss of potency if not carefully formulated or handled. Physical instability, including protein denaturation or aggregation due to temperature changes, further compromises drug effectiveness. Orally administered peptide drugs are especially susceptible to enzymatic and acidic breakdown in the digestive tract, requiring special formulations for oral delivery. Stability is essential for both efficacy and safety, as factors such as temperature, light, and pH can drive degradation pathways that damage the active pharmaceutical ingredient^16^. To overcome these challenges, advanced drug delivery systems, such as polymeric carriers that provide controlled release, hydrogels that protect drugs from degradation, and long-acting formulations like semaglutide, have been developed. These approaches improve drug stability, enhance effectiveness, minimize side effects, and increase patient compliance, offering more promising and practical options for obesity treatment^17^^,^^18^.

### 1.2 Type 2 diabetes (T2D)

T2D is a prevalent chronic disease arising from both genetic and environmental factors^19^. Although its exact pathogenesis is not fully understood, insulin resistance and *β*-cell dysfunction are central mechanisms, often driven by dyslipidemia, hyperglycemia, inflammation, oxidative stress, and ectopic lipid deposition^20^. These factors disrupt glucose homeostasis, resulting in chronic hyperglycemia and metabolic imbalances^19^^,^^20^. If left uncontrolled, T2D significantly increases the risk of complications like retinopathy, nephropathy, neuropathy, heart disease, and cancer^21^. Pharmacological management of T2D focuses on optimizing glycemic control and minimizing complications. Metformin remains the first-line agent due to its ability to improve insulin sensitivity and suppress hepatic glucose production^22^. Sodium-glucose co-transporter 2 (SGLT2) inhibitors promote urine glucose excretion and offer additional cardiovascular and kidney protection^23^. GLP-1RAs, such as semaglutide and liraglutide, not only lower blood glucose but also promote weight loss. GLP-1 is rapidly inactivated by the enzyme dipeptidyl peptidase-4 (DPP-4) because DPP-4 cleaves the peptide bond between the second and third amino acids from the N-terminus, specifically between alanine at position 8 and glutamic acid at position 9 in the GLP-1 sequence, converting it to an inactive form of GLP-1. This cleavage drastically shortens GLP-1’s half-life and limits its insulin-stimulating effects. DPP-4 is widely present on cell surfaces near GLP-1-releasing sites, causing rapid degradation after secretion. Because of this rapid inactivation, DPP-4 inhibitors are used therapeutically to extend GLP-1 activity and improve glucose regulation in T2D, while sulfonylureas increase insulin secretion^24^^,^^25^. Recently, tirzepatide, a dual glucose-dependent insulinotropic polypeptide (GIP) and GLP-1RA (GIP/GLP-1RA), has demonstrated superior efficacy in glycemic and weight management. The treatment regimen is individualized based on the patient’s health status, comorbidities, and treatment goals^26^^,^^27^. Non-pharmacological approaches like exercise, healthy diets, and bariatric surgery are important for managing T2D and related conditions. Among them, bariatric surgery is highly effective for obese T2D patients, improving weight, metabolic health, and comorbidities^28^. Behavioral therapy, probiotics, and good sleep habits can further improve outcomes^28^^,^^29^. Effective T2D care combines medication, lifestyle modification, and psychosocial support^30^. Advanced drug delivery technologies also offer potential benefits in diabetes care, addressing limitations of traditional diabetes therapies, such as poor adherence and injection-related discomfort. These systems aim to provide more effective, long-lasting, and user-friendly ways to administer medication^22^^,^^31^.

To the best of our knowledge, this is the first comprehensive review to focus specifically on the development and application of advanced drug delivery systems for GLP-1RAs in the treatment of obesity and diabetes. This manuscript centers on three major GLP-1RAs: exenatide, liraglutide, and semaglutide, for which these innovative delivery technologies have been most extensively developed and applied. It examines the recent innovations in delivery technologies designed to improve the therapeutic effectiveness, safety, and patient compliance of these agents. The review highlights the drugs’ pharmaceutical and biological properties, explores various delivery systems, and discusses future challenges, including long-term safety, cost-effectiveness, and the need for further clinical validation.

## 2 Overview of glucagon-like peptide-1 (GLP-1), GLP-1 receptor (GLP-1R), and GLP-1RAs

GLP-1, secreted by intestinal enteroendocrine L-cells, regulates post-meal blood glucose levels by stimulating insulin release and suppressing glucagon secretion. It also slows gastric emptying, suppresses appetite, and reduces food intake, optimizing nutrient absorption while helping in weight management^32^. The GLP-1R specifically binds to GLP-1 and exhibits structural and signaling similarities to other class B family receptors (*e.g.*, glucagon, GLP-2, and GIP receptors)^32^^,^^33^. It is widely expressed in tissues such as the pancreas, central nervous system, gastrointestinal tract, and cardiovascular system, highlighting its diverse physiological roles and therapeutic potential beyond glucose regulation^32^^,^^34^^,^^35^. Endogenous (native) GLP-1, whether naturally released or administered as an exogenous peptide, has a very short half-life (≤ 2 min), due to rapid degradation by the DPP-4 enzyme. This limits its effectiveness for obesity and T2D, requiring impractical, frequent administration. GLP-1RAs (also known as GLP-1 agonists, incretin mimetics, or GLP-1 analogs) are structurally modified to resist DPP-4 degradation, which greatly extends their half-life and enhances their therapeutic effects compared to native GLP-1^36^^,^^37^. Examples are exenatide, liraglutide, and semaglutide. While metformin remains the first-line therapy for T2D, GLP-1RAs are recommended for patients with contraindications to metformin (*e.g.*, renal dysfunction, hepatic impairment, heart failure, and lactic acidosis), those with significantly elevated hemoglobin A1c (HbA1c) levels, or those not achieving glycemic control within three months, particularly individuals with conditions such as atherosclerosis, heart failure, or chronic kidney disease. Furthermore, semaglutide and high-dose liraglutide are approved by the FDA for managing obesity^36^^,^^38^^,^^39^. GLP-1RAs have shown promising results in reducing both weight and HbA1c levels, even in individuals with type 1 diabetes (T1D), although their use may be limited by cost and side effects. The 2023 American Diabetes Association guidelines emphasize their cardiovascular advantages, such as reducing major cardiovascular events and potentially slowing the progression of chronic kidney disease. Structurally, GLP-1RAs are classified into human GLP-1 backbone agents (*e.g.*, liraglutide, semaglutide) and exendin-4 backbone agents (*e.g.*, exenatide)^39^. In this review, we will focus on their therapeutic effects on obesity and T2D.

## 3 Mechanisms of GLP-1RAs’ action for obesity and diabetes

GLP-1RAs exert their therapeutic effects on obesity and diabetes through a combination of metabolic and behavioral pathways^40^^,^^41^. In obesity, GLP-1RAs promote weight loss primarily by reducing food intake and increasing satiety, while also improving adipose tissue metabolism and reducing inflammation^42^. Central GLP-1R activation, especially within the hypothalamic ventromedial nucleus, increases energy expenditure and decreases fat accumulation by activating brown adipose tissue (BAT) and promoting the browning of white adipose tissue (WAT) through AMP-activated protein kinase signaling, independent of food intake changes^43^^,^^44^. Adding to this understanding, recent discoveries show that central GLP-1R signaling involves interconnected circuits across the dorsomedial hypothalamus, lateral septum, and hindbrain^45^. GLP-1R neurons in the dorsomedial hypothalamus mediate satiety and feeding behavior by interacting with arcuate nucleus cells, while clustered GLP-1R neurons in the lateral septum are critical for energy homeostasis and drug-induced anorexia^46^. Activation of central GLP-1Rs orchestrates intracellular cascades such as cyclic adenosine monophosphate/protein kinase A and extracellular signal-regulated kinase/mitogen-activated protein kinase across these brain regions, with functional synaptic activity being essential for the full physiological and therapeutic effects of GLP-1RAs^44^^,^^47^.

Clinically, obese patients treated with GLP-1RAs show increased energy expenditure and reduced fat mass, indicating a direct effect in modulating adipose tissue metabolism^48^^,^^49^. GLP-1RAs also reduce inflammation in adipose tissue, which plays a key role in obesity-related metabolic dysfunction^50^. Additionally, central GLP-1 signaling further reduces lipid accumulation in WAT and improves insulin sensitivity, partly through activating the sympathetic nervous system. However, these metabolic benefits are less pronounced in cases of diet-induced obesity, suggesting that obesity may lead to resistance to GLP-1’s effects^51^. These findings highlight the dual action of GLP-1RAs in enhancing BAT activity and reducing adipose tissue inflammation, making them a promising therapeutic option for obesity and its related complications.

In T2D, GLP-1RAs enhance glucose-dependent insulin secretion and suppress glucagon release, improving glycemic control with minimal hypoglycemia risk. These effects are mediated by the activation of cyclic adenosine monophosphate and protein kinase A signaling pathways, as well as glucose metabolism mechanisms. Studies using GLP-1R antagonists or knockout mice have shown that without GLP-1R activation, insulin secretion and glucose tolerance are significantly impaired^39^^,^^52^^,^^53^.

**Figure 1:**
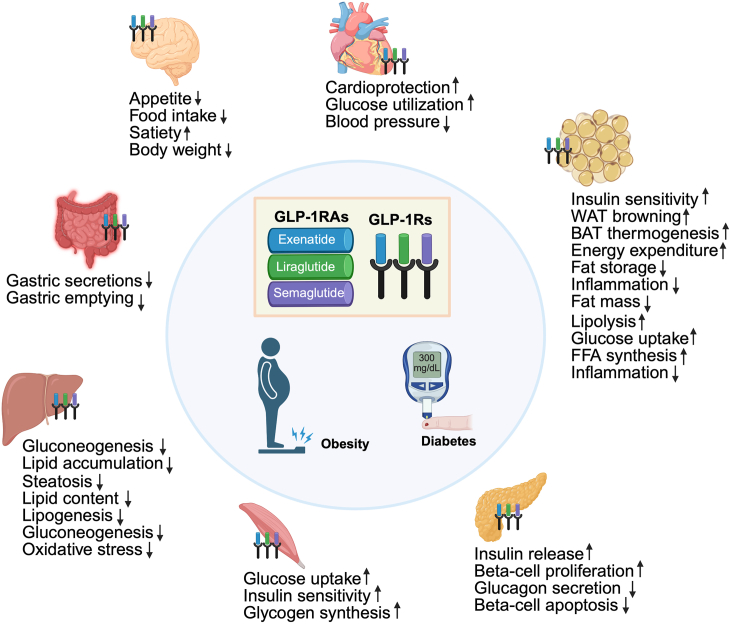
Schematic illustration of GLP-1 receptor agonists (GLP-1RAs), including exenatide, liraglutide, and semaglutide, binding to GLP-1 receptors (GLP-1Rs) in key tissues: brain, gastrointestinal tract, liver, muscle, pancreas, adipose tissue, and cardiovascular system. The diagram highlights tissue-specific effects such as enhanced insulin secretion, appetite suppression, delayed gastric emptying, improved lipid metabolism, increased insulin sensitivity, and cardiovascular protection, illustrating the overall benefits and mechanisms of GLP-1RAs in the treatment of obesity and diabetes.

GLP-1RAs also reduce glucagon secretion indirectly, likely by stimulating somatostatin release from delta (*δ*) cells, since direct GLP-1R expression on human alpha (*α*) cells is minimal. While preclinical studies suggest these drugs help preserve *β*-cell function and mass, clinical trials show that these benefits are limited after discontinuation, possibly due to age-related declines in *β*-cell proliferation and nuclear factor of activated T-cells signaling. In T1D, GLP-1RAs reduce insulin doses and improve insulin sensitivity, but they do not significantly enhance remaining *β*-cell function, indicating their potential as an adjunct therapy rather than a primary therapy^32^^,^^52^. Fig. 1 demonstrates the schematic illustration of GLP-1RAs binding to GLP-1Rs in multiple organs, highlighting their tissue-specific effects and overall benefits for obesity and diabetes.

## 4 Physicochemical properties, pharmacology, pharmacokinetics, and pharmacodynamics of GLP-1 RAs

GLP-1 RAs, including exenatide, liraglutide, and semaglutide, are injectable medications designed for the management of T2D. These agents have undergone structural modifications, such as amino acid substitutions and conjugation with fatty acids or proteins, to extend their half-life. These modifications allow them to resist degradation by DPP-4, reduce renal clearance, and slow down metabolism^54^. Dosing varies among these medications: exenatide is given twice daily or weekly, liraglutide daily, and dulaglutide and semaglutide weekly. Semaglutide is also available as a daily oral tablet, though it has lower bioavailability. GLP-1RAs are administered in different dosages for T2D and obesity to optimize clinical outcomes, specifically, glycemic control for diabetes and weight loss for obesity, while minimizing side effects. Dose titration is important to help reduce common gastrointestinal side effects and enhance efficacy for each condition. Changes in drug absorption, metabolism, and distribution, particularly in patients with obesity, require adjustments to dosage. Safety profiles, patient characteristics (including organ function and body composition), and clinical trial evidence all contribute to developing personalized dosing strategies that ensure both efficacy and safety for each specific disease state^55^. The half-lives range from a few hours for exenatide to several days for dulaglutide and semaglutide, promoting convenient dosing and improving patient adherence^56^. Large peptides have low membrane permeability and a limited distribution, mainly remaining in the plasma and extracellular space^52^. They are primarily eliminated through renal excretion or protein degradation, with minimal liver metabolism^54^. While significant drug–drug interactions are rare, oral medications like contraceptives and levothyroxine may experience delayed absorption, especially with oral or dual agonist formulations. Gradual dose escalation can help reduce gastrointestinal side effects, which are the most common adverse events^57^. Clinical data support their efficacy and safety, with a low risk of hypoglycemia, and ongoing research into the effects of fat mass and kidney function on drug exposure^58^^,^^59^. Below is a summary of the physicochemical properties, pharmacology, pharmacokinetics, and pharmacodynamics of GLP-1 RAs for which advanced delivery methods have been previously studied.

### 4.1 Exenatide

Exenatide (C_184_H_282_N_50_O_60_S; MW around 4186.6 g/moL) is a twice-daily injectable GLP-1RA, typically given in 5–10 μg doses, and shares 53% of its amino acid sequence with native human GLP-1^60^^,^^61^. It is a 39-amino acid peptide that appears as a white powder, soluble in water (approximately 10 mg/mL) and buffers. Although it does not have a defined melting point, it decomposes when heated^62^. Derived from Gila monster venom, it is resistant to DPP-4 degradation, resulting in a significantly extended half-life compared to native GLP-1, which lasts 1.5 to 2 min. Its half-life varies by formulation: about 2.4 h for Byetta, which requires twice-daily dosing, and 5 to 6 days for Bydureon. Bydureon, encapsulated in biodegradable microspheres, allows for once-weekly dosing with sustained plasma levels maintained for several weeks^52^^,^^63^^,^^64^. Immediate-release exenatide (Byetta) starts at 5 μg subcutaneously twice daily, increasing to 10 μg twice daily for T2D. Extended-release exenatide (Bydureon) is given as a 2 mg subcutaneous injection once weekly. Pharmacokinetic studies show that, following subcutaneous injection, exenatide has a bioavailability of 65%–75%, a volume of distribution of around 28.3 L, reaches peak plasma levels in about 2.1 h, and binds minimally to plasma proteins^65^. Its absorption follows a two-compartment model with first-order kinetics, and elimination occurs *via* both linear and nonlinear pathways. Exenatide is mainly metabolized by proteolysis and excreted primarily through glomerular filtration, so dose adjustments are required for patients with impaired kidney function^63^. Clinical studies have shown that exenatide lowers HbA1c by 0.8% to 1.1% over 30 weeks, primarily through glucose-dependent insulin secretion, suppression of glucagon, delayed gastric emptying, and appetite reduction. These effects, along with modest weight loss of 1.6 to 2.8 kg, highlight its effectiveness in managing obesity and T2D^66^, ^67^, ^68^.

### 4.2 Liraglutide

Liraglutide, a once-daily GLP-1RA that shares 97% amino acid similarity with endogenous human GLP-1 but has been structurally modified to enhance resistance to enzymatic degradation and prolong its activity^69^^,^^70^. It is a white powder (C_172_H_265_N_43_O_51_; MW 3751.20 g/mol) that is freely soluble in aqueous basic solutions (>270 mg/mL) but shows markedly reduced water solubility below pH 7, reaching its lowest (around 0.05 mg/mL) near its isoelectric point of 4.9. It has a melting point above 182 °C, where it decomposes^71^. Key modifications include the attachment of a palmitic acid (C16 fatty acid) *via* a glutamic acid spacer to the lysine at position 26 and substituting arginine for lysine at position 34. These changes enhance liraglutide’s binding to albumin (over 98%) and decrease its susceptibility to DPP-4 degradation^72^^,^^73^. These modifications increase liraglutide’s half-life to about 13 h, compared to just 1.5–2 min of native GLP-1, allowing for once-daily subcutaneous administration. Therapy usually begins at 0.6 mg subcutaneously once daily and is gradually increased to maintenance doses of up to 1.8 mg for T2D and up to 3 mg for obesity^74^.

Pharmacokinetically, liraglutide has a bioavailability of about 55% after subcutaneous injection and a volume of distribution between 13 and 25 L. It reaches peak plasma concentration (*T*_max_) within 8 to 14 h, with over 98% bound to plasma proteins, mainly albumin. It is metabolized through proteolytic pathways without minimal involvement of the liver or kidneys, so dose adjustments are not needed for patients with hepatic or renal impairment^72^^,^^75^^,^^76^.

Clinical trials have shown that liraglutide reduces HbA1c by 0.8% to 1.5% and leads to a weight loss of 2 to 3 kg over 26 weeks^74^. Its glucose-dependent mechanism stimulates insulin secretion from pancreatic *β*-cells and suppresses glucagon release, helping to reduce hyperglycemia without increasing the risk of hypoglycemia. Liraglutide also slows gastric emptying, promotes satiety, and improves cardiovascular risk factors such as body weight, blood pressure, and lipid profiles, through central nervous system effects. Clinical studies indicate liraglutide has minimal drug–drug interactions, although its effect on gastric emptying may temporarily alter the absorption of oral medications. Approved for both T2D and obesity, liraglutide’s sustained efficacy is due to its balanced pharmacokinetic and pharmacodynamic profile, which combines extended receptor activation with metabolic stability^69^^,^^77^.

### 4.3 Semaglutide

Semaglutide (C_187_H_291_N_45_O_59_; MW around 4113.58 g/mol), a once-weekly GLP-1RA with 94% amino acid similarity to human GLP-1, is a white to off-white powder exhibiting moderate water solubility of approximately 0.9 mg/mL at acidic pH, poor solubility near neutral pH; it thermally decomposes above 200 °C^78^. Semaglutide can be given as a once-weekly subcutaneous injection, starting at 0.25 mg and increasing to 2.4 mg for obesity or 2 mg for T2D. Alternatively, it can be taken as a once-daily oral tablet, starting at 3 mg and increasing to 14 mg based on response and tolerability. Structural modifications, including the addition of a fatty acid side chain, promote strong albumin binding and resistance to DPP-4 degradation, resulting in a significantly extended half-life of about 7 days, compared to just 1.5–2 min for native GLP-1^6^^,^^79^. Pharmacokinetically, Semaglutide has a once-weekly subcutaneous dosing option with a high bioavailability of about 89%, reaching peak plasma concentration within 1 to 3 days. It is metabolized through proteolytic cleavage and *β*-oxidation, and is excreted in urine and feces without hepatic metabolism^6^. Oral semaglutide is combined with salcaprozate sodium, which enhances its stomach absorption. SNAC raises the local pH to protect semaglutide from gastric enzymes, aids in monomerization, and temporarily fluidizes gastric membranes for better absorption. Consequently, semaglutide has a low bioavailability of about 0.8%, affected by fasting and water intake. Although absorbed faster than the subcutaneous form, it has similar distribution, metabolism, and half-life characteristics. Once-daily oral dosing has steady-state exposure in 4 to 5 weeks with reduced variability^80^^,^^81^. Both formulations show dose proportionality and a consistent safety profile^81^.

Clinically, semaglutide lowers HbA1c by 1.5% and reduces body weight by 5.6 kg over 56 weeks, acting through mechanisms such as glucose-dependent insulin secretion, suppression of glucagon by about 30%, delayed gastric emptying (50% slower), and appetite regulation *via* hypothalamic GLP-1R^82^^,^^83^. Semaglutide’s ability to promote significant weight loss of up to 15% in obesity trials and decrease cardiovascular risk highlights its dual benefit in T2D and long-term weight management^84^^,^^85^. The most common side effects are gastrointestinal, with nausea reported in 20%–40% of patients, while injection-site reactions are less frequent (1.2%) compared to other GLP-1RAs^84^^,^^86^^,^^87^.

## 5 Adverse effects of GLP-1RAs

GLP-1 RAs are normally well tolerated. However, they commonly cause gastrointestinal side effects, especially during initiation of administration or dose escalation. The incidence of nausea varies among these medications: 20.4% for liraglutide, 9.4% for exenatide, and about 20% for semaglutide. Diarrhea occurs in 13.1% of liraglutide users, 6.1% of those on exenatide, and 12% of semaglutide users, while vomiting is reported in 10.7%, 3.7%, and 9% of users, respectively^88^, ^89^, ^90^. Exenatide, especially in its once-weekly formulation, is associated with more injection site reactions than daily liraglutide or semaglutide. Despite initial concerns about pancreatitis risk, recent studies indicate that GLP-1RAs may not significantly increase this risk and could even reduce it in some populations^91^. However, patient monitoring is recommended for rare but serious adverse events such as pancreatitis and gallbladder disorders, which are more frequent with liraglutide (6.2%) and semaglutide (9.8%) compared to placebo. Additionally, these medications may also cause a slight increase in resting heart rate, but most cardiac arrhythmias observed are non-serious^90^. All three medications can cause hypoglycemia, especially when combined with sulfonylureas. Regarding pharmacokinetics, Liraglutide maintains steady plasma levels over 24 h, while exenatide peaks and returns to baseline within 10–12 h, and semaglutide offers a long half-life that supports once-weekly dosing, affecting dosing schedules and efficacy^6^^,^^75^. Clinically, while some approaches have been tried to help manage or reduce these side effects, they have been demonstrated not to eliminate them entirely.

## 6 Challenges in traditional delivery methods for GLP-1RAs

Oral administration is the preferred route for drug delivery due to its convenience and high patient compliance. However, delivering GLP-1RAs peptides orally presents significant challenges. The acidic pH and enzymatic activity in the gastrointestinal environment can degrade these peptides before absorption. Moreover, the presence of mucus and epithelial barriers in the intestine limits the absorption of these large, hydrophilic molecules^92^^,^^93^. As a result, the bioavailability of oral formulations is extremely low, accounting for less than 1% of the GLP-1RA market^94^.

Although subcutaneous injection remains the primary method for administering GLP-1RAs like liraglutide and exenatide, it poses challenges for patient adherence and convenience. Particularly, these GLP-1RAs require frequent administration due to their relatively short half-lives, which can be inconvenient for patients^95^^,^^96^. Liraglutide requires daily injections, while exenatide is available in both twice-daily and once-weekly formulations. The weekly exenatide formulation requires mixing a diluent with the powdered medication before injection, making the preparation step complicated and potentially inconvenient for patients. Consequently, the frequent and sometimes complex dosing regimens could lead to decreased adherence, especially among individuals with needle aversion or who view regular injections as disruptive to their daily lives^97^^,^^98^. A study comparing liraglutide and exenatide found that adherence to treatment was higher for liraglutide, with a 6%–7% loss of adherence over 16 months, compared to a 10%–13% loss for exenatide. This difference may be due to liraglutide’s once-daily dosing, which is generally easier for patients to manage than the twice-daily regimen required for exenatide^99^. Additionally, administering these medications subcutaneously can result in injection-site reactions such as rash, redness, itching, and, in some cases, nodules, which may further reduce patient compliance and impact the overall effectiveness of the treatment^100^. GLP-1RAs can cross the blood–brain barrier (BBB) primarily through active, receptor-dependent transcytosis that involves GLP-1 receptors located on brain endothelial cells. This process requires the binding of the ligand and subsequent activation of intracellular signaling. Due to the peptide nature and size of these molecules, passive diffusion is limited. Importantly, the ability of different GLP-1RAs to penetrate the BBB varies^101^^,^^102^. For instance, exenatide crosses the BBB more readily than liraglutide or semaglutide, likely due to differences in their molecular structures and transport mechanisms^103^. Traditional methods for delivering GLP-1RAs, such as subcutaneous injections or oral administration, face significant challenges in achieving effective concentrations in the brain because of the restrictive nature of the BBB. This barrier limits the passage of these drugs mainly because GLP-1RAs are relatively large, hydrophilic peptides with low lipid solubility, which hinders passive diffusion^45^^,^^102^. Mechanistically, GLP-1RAs may cross the BBB through slow adsorptive-mediated transcytosis or passive diffusion at circumventricular organs, which lack strong BBB protection. However, the concept of receptor-mediated transcytosis *via* the GLP-1 receptor on endothelial cells remains a topic of debate due to the low expression of these receptors. As a result, the combination of traditional dosing methods and the limitations imposed by the BBB can restrict drug levels in critical brain regions, thereby limiting the therapeutic effects of these agents within the central nervous system^52^^,^^102^.

Recent advances have led to the development of orally available small-molecule GLP-1RAs that aim to overcome the limitations of traditional delivery methods for GLP-1RAs. Small molecules such as danuglipron, aleniglipron (GSBR-1290), MLX-7005, ID110521156, and RGT075 have been developed, demonstrating promising efficacy in promoting insulin secretion, reducing appetite, and facilitating weight loss. Clinical trials with danuglipron and aleniglipron have shown dose-dependent improvements in glucose tolerance, significant reductions in HbA1c and body weight, and generally mild to moderate gastrointestinal side effects largely occurring during dose escalation. These compounds offer distinct advantages, including improved tissue permeability, extended half-lives, oral bioavailability, and potentially lower production costs compared to peptide-based GLP-1RAs. However, Challenges remain in optimizing oral administration and reducing gastrointestinal side effects, which can impact patient compliance. Therefore, further research is needed to better understand the efficacy and safety of these agents before they can be widely used in treating obesity and diabetes^104^^,^^105^. MariTide (AMG 133) is another advanced formulation that represents a promising next-generation therapy for obesity^106^. It is a bispecific molecule that combines a GIP receptor antagonist antibody with two GLP-1RA peptides, designed for once-monthly subcutaneous injection. This dual-action approach offers potentially greater and more sustainable weight loss compared to traditional GLP-1RAs. Clinical studies demonstrated up to 20% average weight loss at 52 weeks without a plateau, alongside significant improvements in metabolic health^107^. However, despite its potential, the injectable delivery route has limitations, including the possibility of gastrointestinal side effects and the need for more long-term safety and efficacy data^106^^,^^108^.

Consequently, developing alternative delivery systems, such as nano- and micro-carrier-based delivery systems, hydrogels, transdermal patches, and enhanced longer-acting or combined formulations, could help overcome challenges and improve patient adherence to GLP-1RA therapies^93^^,^^109^^,^^110^. Below, we introduce some novel delivery systems that have been developed most recently.

## 7 Advanced drug delivery systems (DDS)

### 7.1 Nanocarrier drug delivery systems (NDDS)

NDDS are advanced drug delivery systems developed at the nanoscale (1–1000 nm) to improve the targeted delivery and controlled release of therapeutic agents. By encapsulating or conjugating drugs within nanostructures capable of penetrating biological barriers, these systems are designed to increase drug efficacy, enhance bioavailability, and reduce side effects by delivering therapeutic agents directly to specific tissues or cells^111^. For GLP-1RAs, NDDS can address BBB limitations by protecting drugs from enzymatic degradation, prolonging circulation time, and enabling surface modifications with targeting ligands to facilitate active transport across the BBB^102^^,^^112^. Cationic surface engineering further promotes adsorptive-mediated uptake, while controlled release formulations help maintain stable CNS drug concentrations^113^. Additionally, nose-to-brain delivery with nanocarriers can bypass the BBB entirely, enabling direct, noninvasive access to the brain, as demonstrated in preclinical GLP-1RA studies^114^. A variety of nanocarriers have been developed for drug delivery, including dendrimers, liposomes, solid lipid nanoparticles (NPs), polymersomes, polymer–drug conjugates, polymeric NPs, peptide NPs, micelles, nanoemulsions, nanospheres, nanocapsules, nanoshells, carbon nanotubes, and gold NPs. Each offers unique advantages^115^^,^^116^. *Dendrimers* are hyperbranched, nanoscale molecules that can encapsulate or bind drugs, enhancing their solubility and stability through both covalent and non-covalent interactions^116^^,^^117^. *Liposomes*, composed of lipid bilayers, can carry both hydrophilic and hydrophobic drugs, and are valued for their biocompatibility, low immunogenicity, and ability to be engineered for targeted and sustained release. They are the most widely used nanocarriers, with several liposomal drugs approved for clinical use^116^^,^^118^. *Solid lipid NPs* are lipid-based carriers that remain solid at body temperature. They provide drug protection and enable controlled release; however, their crystalline structure can limit drug loading and cause burst release^116^^,^^119^. *Polymersomes*, which are formed through the self-assembly of amphiphilic block copolymers, can encapsulate both hydrophilic and hydrophobic drugs. Their properties can be adjusted for various biomedical applications^116^^,^^120^. *Polymeric NPs*, derived from natural or synthetic polymers, facilitate targeted and stimuli-responsive drug delivery; however, their clinical application remains limited^116^^,^^121^. *Peptide NPs* self-assemble to deliver therapeutic peptides effectively for drug delivery and imaging purposes^116^^,^^122^. *Micelles* are structures with both hydrophilic and hydrophobic properties that enhance the solubility and targeting of hydrophobic drugs, facilitating sustained release and deep tissue penetration^116^^,^^123^. *Nanoemulsions*, which consist of nano-sized oil or water droplets, enhance the delivery of poorly soluble drugs and can be customized for site-specific therapies^124^^,^^125^. *Nanospheres* are matrix-structured polymeric particles that protect drugs and enable targeted release but may aggregate or be rapidly cleared^116^. *Nanoshells*, especially gold-based, are used in imaging, targeted therapy, and photothermal applications due to their stability and biocompatibility^126^. *Carbon nanotubes* are hollow structures with a high capacity for drug loading and the ability to penetrate cells; however, they require functionalization for safety and compatibility, and their health risks must be carefully assessed^127^. *Gold nanoparticles* are valued for their optical properties, which enable targeted drug delivery, imaging, and photothermal therapy. However, their safety and environmental impact must be addressed^116^^,^^128^. Antibody drug conjugates are a novel treatment option for various chronic conditions. Comprising monoclonal antibodies linked to therapeutic agents, antibody drug conjugates allow for precise targeting and controlled drug release, improving efficacy and reducing side effects by delivering potent drugs directly to specific cells. Their nano-scale size makes them effective for protein and peptide therapeutics^129^^,^^130^. However, no antibody drug conjugates have yet been developed for GLP-1RAs.

These NDDS have several medical applications in cancer therapy, cardiovascular disease treatment, neurodegenerative disorders, regenerative medicine, medical imaging, gene therapy, immunotherapy, and metabolic disease management^131^. In obesity, nanocarriers offer promising solutions by facilitating targeted delivery to adipose tissue, stimulating thermogenesis, modulating inflammatory cells, and selectively delivering therapeutic agents to improve metabolic health. This targeted approach has the potential to result in more effective treatments with fewer side effects compared to conventional therapies^132^, ^133^, ^134^. Biocompatible and biodegradable materials, such as natural polymers (*e.g.*, alginate, gelatin, albumin), synthetic polymers (*e.g.*, polylactides), and lipids, have been used for the synthesis of NDDS^111^. These materials enable controlled drug release through mechanisms such as diffusion, swelling, erosion, or matrix degradation. Some nanocarriers respond to specific stimuli, like pH or temperature, resulting in controlled drug release, which is beneficial for targeted treatments such as cancer therapy^135^^,^^136^.

NDDS significantly improves drug stability, bioavailability, and efficacy, including GLP-1RAs for obesity and diabetes treatment^137^^,^^138^. Despite the many advantages of nanocarriers in drug delivery systems, several significant limitations remain. Ensuring safety and biocompatibility is challenging, as ideal nanocarriers must be non-toxic, biodegradable, and able to evade immune detection, a combination that is difficult to achieve and requires extensive research and testing. Scaling up production while maintaining consistent quality is also complex and costly, with variations potentially affecting efficacy and safety. Regulatory approval is further complicated by the lack of standardized assessment protocols and the need for robust characterization methods and safety guidelines. Nanocarriers must also remain stable in biological environments until they reach their target, as instability can lead to premature drug release or degradation. Effective targeting of specific cells or tissues without off-target effects remains a major hurdle, and some nanocarriers suffer from short half-lives or low solubility, limiting their therapeutic potential. Despite these challenges, advances in materials science and nanotechnology continue to drive progress, but overcoming issues related to safety, scalability, regulation, and stability is essential for successful clinical translation^116^. In this review, we will discuss the development and application of various nanocarrier systems for enhancing the delivery of GLP-1RAs and other therapeutics in metabolic disease management.

### 7.2 Microsphere and microparticle systems

Drug-loaded microspheres and microparticles are advanced technologies in drug delivery systems, overcoming many limitations of traditional methods^139^. *Microspheres* are spherical, free-flowing particles that can be either organic or inorganic. They have diameters ranging from 1 to 1000 μm. Their high surface area-to-volume ratio is particularly beneficial for controlled and sustained drug release, which enhances the efficacy and targeting of the encapsulated agents^140^. Microspheres are distinct for their ability to retain shape and structural integrity even when loaded with drugs or combined with other materials. Their porous three-dimensional structure makes them valuable in tissue engineering, serving as scaffolds to support cell growth and regeneration^141^^,^^142^. Microspheres can be engineered for various biomedical functions, including cell encapsulation and adhesion. They are commonly classified into two categories: microcapsules and microcarriers. Microcapsules have a core–shell structure that traps cells or active ingredients, while microcarriers provide surfaces that promote cell adhesion. The typical size range for these applications is between 100 and 400 μm, which is optimized to support oxygen diffusion and maintain cell viability. Depending on the intended therapeutic target, microspheres can be customized for specific mechanical strength and porosity, allowing for targeted delivery to particular cell types or tissues^139^^,^^140^^,^^143^. *Microparticles* include both microspheres and microcapsules and can be made from a wide range of materials, such as polymers, ceramics, glass, metals, and composites^139^^,^^144^. They come in various structural forms, including microgranules, micropellets, microsponges, microemulsions, magnetic microparticles, and lipid vesicles (such as liposomes and noisomes)^145^. Microparticles can be solid, porous, or hollow^146^. Polymeric microparticles, especially those made from biodegradable or synthetic polymers, are widely used due to their versatility and biocompatibility^147^. Microsphere and microparticle delivery systems provide controlled and prolonged drug release, which can help reduce the frequency of dosing. Furthermore, these systems minimize toxicity, improve bioavailability, and support sustained CNS exposure, potentially improving drug penetration when the BBB’s permeability is transiently increased, and enhancing the solubility of poorly soluble drugs. These systems also protect drugs from degradation, mask unpleasant tastes or odors, and enable targeted delivery, all of which enhance patient compliance^141^. Microspheres and microparticles can be fabricated using methods like emulsion-solvent extraction, precision particle fabrication, thermally induced phase separation, and spray drying^139^. Spray drying is ideal for creating dry powders and granules with narrow particle size distributions^139^. It effectively microencapsulates sensitive substances like volatiles and probiotics into uniform, micron-sized particles in a single step. Despite some drawbacks, such as material loss and low yield, it is particularly useful for encapsulating heat-sensitive materials. Moreover, extruded wax particles can congeal to embed hydrophilic components for sustained release through slow erosion^139^^,^^148^.

Despite their many advantages, microsphere and microparticle systems face certain limitations, including complex and costly manufacturing processes, potential batch-to-batch variability, and environmental concerns related to the degradation of some components^141^^,^^149^. Achieving a uniform pore structure, consistent drug loading, and reproducible release profiles is a significant technical challenge. However, continuous advancements in materials science and fabrication technologies are driving innovation in this field. As a result, many new microparticulate products are currently in clinical trials or already available on the market^150^. Liraglutide and Exenatide-loaded microspheres have been previously developed and applied for obesity and diabetes, which showed improved tolerability and effectiveness. This review summarizes advancements in GLP-1RAs-loaded microsphere and microparticle delivery systems with potential applications for treating obesity and diabetes.

### 7.3 Hydrogel-based delivery systems

Hydrogel-based delivery systems are highly valued in clinical applications for their capacity to provide controlled and localized release of therapeutic agents. These systems are composed of highly hydrated polymer networks, formed from natural, synthetic, or semi-synthetic polymers, that are crosslinked either physically or covalently. This unique structure allows hydrogels to efficiently encapsulate and protect a wide range of bioactive substances, from small molecules to proteins and nucleic acids, while maintaining excellent biocompatibility and customizable physical and chemical properties^151^^,^^152^.

One major benefit of hydrogels is their capability to provide precise spatiotemporal control over drug release, enabled by advances in polymer chemistry, bioengineering, and nanotechnology^153^. Hydrogels can be designed to respond to specific environmental stimuli, such as pH, enzymes, or temperature, allowing for precise control over the timing and dosage of drug release at the target site^152^. Their adaptability is further improved by tailoring biocompatibility, mechanical strength, and degradability through various crosslinking strategies^154^. For BBB delivery, hydrogels offer unique advantages as they mimic the extracellular matrix of the CNS, facilitating drug diffusion and providing a supportive scaffold that minimizes tissue disruption and immune response. However, challenges remain, including limited penetration depth and drug diffusion variability within brain tissue, which can affect uniform distribution and retention. Combining hydrogels with nanoparticles and stimulus-responsive functionalities can further improve BBB penetration, drug stability, and targeted delivery efficacy^155^^,^^156^.

Hydrogels can be classified into three main categories: natural, synthetic, and semi-synthetic. Natural hydrogels consist of materials like chitosan, alginate, fibrin, gelatin, and hyaluronic acid. On the other hand, synthetic hydrogels are usually made from polymers such as poly(ethylene glycol) (PEG) and poly(vinyl alcohol). Semi-synthetic hydrogels, like gelatin methacryloyl, combine natural components with synthetic functional groups, which enhances their versatility. These materials have been extensively studied for various applications, including cancer therapy, infection treatment, wound healing, and tissue engineering^157^. Hydrogel delivery systems are suitable for various routes of administration, including oral, local parenteral (*in situ* implantation), topical/transdermal, and ocular delivery. Their versatility, combined with the ability to protect and release a wide range of therapeutic agents in a controlled manner, underscores the significant potential of hydrogel-based systems in enhancing drug delivery and advancing regenerative medicine^158^. Oral hydrogels have significant limitations compared to injectable hydrogels, including poor permeability across the gastrointestinal lining, potential enzymatic degradation before absorption, and variable conditions in the GI tract that affect stability and bioavailability. They also generally have lower mechanical strength and challenges with sustained release. However, stimuli-responsive hydrogels play a vital role in oral delivery as they can respond to environmental changes. These hydrogels can be triggered by various physical and chemical stimuli, such as pH, light, ionic strength, solvent composition, temperature, and electric fields, enabling controlled and targeted drug release in the complex GI environment. In contrast, injectable hydrogels offer precise, localized delivery with sustained release and better stability while bypassing first-pass metabolism. However, they require invasive administration, which can lead to injection site reactions and compliance issues^159^^,^^160^. Despite their potential, hydrogels also face technical challenges that limit their clinical use, including difficulties in precisely controlling gelation, concerns about immunogenicity, and obstacles in large-scale production. Ongoing research aims to address these issues by developing new crosslinking methods, improving hydrogel stability, and ensuring safe biodegradation after treatment. Furthermore, the integration of nanocarriers into hydrogels is being investigated to increase drug loading and improve release profiles, thus expanding the therapeutic applications of these systems^158^^,^^161^.

Hydrogel-based delivery systems have been developed to provide sustained, long-acting release of GLP-1RAs, enabling month-long therapeutic exposure from a single injection. This approach has shown promise in preclinical models, maintaining effective blood glucose and weight management with far less frequent dosing compared to daily or weekly injections, and has the potential to significantly lower the frequency of therapeutic interventions, thereby greatly enhancing patient quality of life^161^, ^162^, ^163^. This review explores applications and highlights recent advances and future directions in the hydrogel-based delivery of GLP-1RAs.

### 7.4 Microneedle (MN) technology for transdermal delivery

Transdermal Drug Delivery Systems (TDDS) are advanced, non-invasive techniques that deliver medication through the skin. These systems provide several advantages, such as avoiding gastrointestinal side effects, bypassing first-pass metabolism, and maintaining consistent plasma drug levels. TDDS are convenient, painless, and ideal for long-term therapy, which improves patient compliance and minimizes adverse effects. They are widely used in pharmaceuticals and cosmetics, with various disease management to skincare applications^164^^,^^165^. MNs are minimally invasive devices designed to penetrate the skin’s outermost layer, the stratum corneum, to enhance drug delivery. These micron-scale needles, typically 25–2000 μm in length, create microchannels that allow efficient drug absorption without causing significant pain. Advances in materials like polymers, silicon, and metals have further improved MN technology. MN systems offer distinct advantages over other DDS, combining ease of self-administration, sustained drug release, and reduced dosing frequency, which are especially beneficial for chronic diseases like obesity and T2D. Unlike NPs, microparticles, and hydrogels, MNs enable precise, targeted delivery directly through the skin to adipose tissue, enhancing therapeutic effects such as fat browning and glycemic control at lower doses. This localized delivery reduces systemic exposure, minimizing side effects. Additionally, MNs provide controlled and consistent drug release, improving treatment efficacy and patient adherence by overcoming barriers associated with injections or oral medications. Furthermore, MNs can be integrated with other delivery systems, especially NPs and microparticles, to combine their advantages, such as enhanced drug stability, targeted delivery, and controlled release, thus further optimizing therapeutic outcomes. These combined benefits make MN technology an efficient, safer, and more patient-friendly alternative for managing chronic metabolic conditions^116^^,^^166^. Solid MNs enhance drug absorption by creating microchannels in the skin. Hollow MNs inject drug solutions directly into the tissue, allowing for precise dosing and rapid effects. Coated MNs deliver drugs through surface dissolution, making them suitable for administering low-dose drugs. Dissolving MNs release drugs as they gradually degrade, reducing disposal risks. Swelling MNs absorb bodily fluid to modulate drug release, enabling sustained delivery^167^. MNs are fabricated using materials like polymers, metals, silicon, and ceramics, with polymers (*e.g.*, PLGA, hyaluronic acid) favored because of their biocompatibility. Natural materials like polysaccharides (*e.g.*, cellulose, chitosan) and proteins (*e.g.*, gelatin, collagen) are also explored for their excellent biocompatibility and minimal irritation. Fabrication methods include micro-molding, 3D printing, and laser techniques, enabling precise control over MN geometry and drug loading^168^.

MNs have great potential for drug delivery, but they do face several limitations. One major issue is their limited loading capacity, which reduces the total amount of drug that can be loaded/delivered. Additionally, polymeric MNs may sometimes lack sufficient mechanical strength to penetrate the skin effectively. Variations in individual skin properties can also affect the depth and consistency of MN insertion^169^. There is also a lack of clear regulatory guidelines and limited industry investment, which complicates the development and implementation of MN technology. Despite challenges, MNs offer pain-free and sustained delivery of therapeutics like GLP-1 agonists, enhancing the management of chronic conditions. This review will explore these applications, highlighting recent advances and future directions in GLP-1RAs MN-mediated delivery^169^^,^^170^.

### 7.5 Other enhanced drug formulations

Recent advancements in drug formulation have led to the development of enhanced delivery systems, such as long-acting (extended-release) and novel co-formulations, which are transforming modern medicine. Long-acting formulations ensure sustained drug release, improve patient compliance, and reduce dosing frequency. Meanwhile, innovative co-formulations combine multiple active agents, such as biologics or a biologic with a small molecule, into a single dosage form to target multiple disease pathways and optimize therapeutic outcomes. These advancements are supported by cutting-edge technologies like nanotechnology, which enables targeted delivery and improved bioavailability, allowing for personalized and complex drug structures. These advancements are resulting in safer, more effective, and patient-focused treatments, marking a new era in personalized medicine and enhanced care quality^171^.

#### 7.5.1 Long-acting drug delivery formulations (LADDS)

Traditional pharmaceuticals are typically designed as short-acting agents that require frequent administration. This can lead to poor patient adherence and increase the risk of inconsistent therapeutic outcomes. In contrast, LADDS are engineered to release medications steadily over extended periods, ranging from weeks to years. This approach enhances patient compliance and therapeutic efficacy, particularly for managing chronic conditions^172^^,^^173^. LADDS use a variety of advanced technologies and materials to achieve controlled and prolonged drug release^172^. These methods include dissolution-based systems, biodegradable implants (both preformed and *in situ*-forming), non-degradable implantable devices, and hydrogel-based formulations^173^. Such systems have been applied clinically in areas like long-acting contraceptives, hormone suppression therapies, treatments for opioid and alcohol dependence, and localized ocular drug delivery^174^. Importantly, the U.S. FDA has approved over 60 long-acting drug products that maintain consistent drug levels for a month or longer, underscoring their growing significance in modern medicine^174^. The mechanisms of these formulations vary: some include gradual drug dissolution, others rely on slow polymer degradation, and some use sustained diffusion from hydrogels or non-degradable implants. These methods reduce the rapid fluctuations in drug concentration seen with immediate-release formulations, minimizing side effects and improving therapeutic outcomes^174^^,^^175^. LADDS can be delivered *via* oral sustained-release tablets, injectables, implants, transdermal patches, microspheres, or nanoparticles^173^^,^^176^. They are especially valuable for chronic disease therapies, including pain, cancer, central nervous system disorders, and diabetes, by providing more stable and effective long-term treatment^176^. Despite their benefits, LADDS face challenges such as high material costs, limited biomaterial availability, system complexity, and reliance on external triggers for some formulations. Regulatory barriers and a lack of suitable excipients for new technologies like 3D printing and microneedles also hinder development and approval^172^^,^^173^^,^^177^. LADDS for GLP-1RAs have shown significant effectiveness in preclinical and clinical studies, improving metabolic outcomes in obesity and fatty liver disease, as well as offering prolonged glycemic control in diabetes^178^, ^179^, ^180^. Clinical trials of once-weekly exenatide have demonstrated better glucose regulation and weight loss in T2D, emphasizing the potential of these systems to enhance treatment efficacy and patient adherence^180^.

#### 7.5.2 Co-formulation approaches in advanced drug delivery

Co-formulation is an innovative drug delivery strategy that combines two or more therapeutically active agents, such as biologics and small molecules, into a single formulation. This method strengthens therapeutic effects, enhances formulations, and targets multiple disease pathways simultaneously ^181^^,^^182^. Recent advancements in formulation technologies, such as micro- and nano-encapsulation, three-dimensional (3D) printing, and controlled-release devices, have facilitated the creation of co-formulated products for both local and systemic delivery^181^^,^^183^. Co-formulated therapies are used in diabetes (*e.g.*, combinations of short- and long-acting insulin), oncology (antibody cocktails), and respiratory diseases (inhalers with bronchodilators and corticosteroids). These products enhance efficacy through synergy and offer extended intellectual property protection for manufacturers^181^^,^^184^^,^^185^. However, co-formulation presents significant challenges. Technical hurdles include ensuring the stability and compatibility of multiple agents, optimizing pharmacokinetics and pharmacodynamics, and preventing unwanted interactions, such as protein aggregation or degradation. Regulatory pathways are also more complex, as co-formulated products must demonstrate safety and efficacy for each component, both individually and in combination, often requiring additional clinical trials. Analytical challenges arise in quality control, where sensitive methods are needed to distinguish and monitor each component and its degradation products^181^^,^^186^.

Despite these challenges, ongoing innovations, such as the use of excipients like hyaluronidase to enhance subcutaneous absorption or the application of 3D printing for personalized combination therapies, are paving the way for broader clinical translation. Ultimately, the success of co-formulation strategies will depend on balancing technical feasibility, regulatory requirements, and clear clinical benefit for patients^181^^,^^182^.

**Figure 2:**
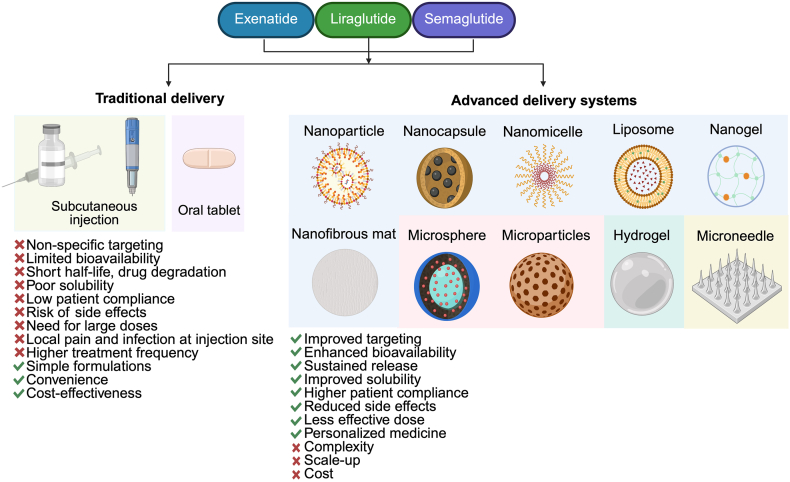
Comparison of conventional and advanced GLP-1 receptor agonist (GLP-1RA) delivery systems, summarizing their respective benefits and limitations in terms of efficacy, safety, patient compliance, and targeted delivery.

Advancements in GLP-1 formulations include novel co-formulations combining basal insulin with GLP-1RAs, such as IDegLira (insulin degludec/liraglutide) and iGlarLixi (insulin glargine/lixisenatide). These synergistic combinations improve glycemic control and reduce side effects compared to individual therapies. Clinical trials confirm their effectiveness in insulin-naive and inadequately controlled patients^187^. Additionally, nano-formulations of GLP-1RAs combined with SGLT2 inhibitors show promise in improving lipid metabolism, offering comprehensive benefits for T2D management^188^. Fig. 2 demonstrates the comparative overview of traditional and advanced formulations and delivery systems for GLP-1RAs, highlighting their respective advantages and disadvantages in terms of efficacy, safety, patient compliance, and targeted delivery.

## 8 Advanced delivery systems for GLP-1RAs delivery in obesity and diabetes

The development of novel drug delivery systems represents a multidisciplinary advancement that combines discoveries from biology, physiology, pharmacology, chemical engineering, and pharmaceutical sciences. By employing advanced technologies and methodologies, these systems effectively address key limitations of traditional drug delivery, such as poor bioavailability, inconsistent plasma drug levels, and the inability to achieve sustained release^22^^,^^116^. Among the most promising strategies for treating obesity and diabetes are advanced drug delivery systems such as nano-, micro-carriers, hydrogels, MNs, and other enhanced formulations such as long-acting and novel co-formulations. NDDS enhances the targeted delivery and controlled release of GLP-1RAs like exenatide, liraglutide, and semaglutide by protecting these agents from enzymatic degradation, improving their bioavailability, and enabling sustained release. Microsphere and microparticle systems further facilitate prolonged drug action and reduce dosing frequency, while hydrogel-based platforms provide a biocompatible matrix for extended and responsive drug release. MN technology offers a minimally invasive, painless, and efficient method for transdermal administration, increasing patient comfort and adherence. In addition, long-acting formulations and innovative co-formulations, such as combinations of GLP-1RAs with other therapeutic agents, address multiple disease pathways, optimize therapeutic outcomes, and simplify treatment regimens. Collectively, these advanced delivery technologies are transforming the management of metabolic diseases by improving efficacy, safety, and patient experience^189^, ^190^, ^191^.

**Figure 3:**
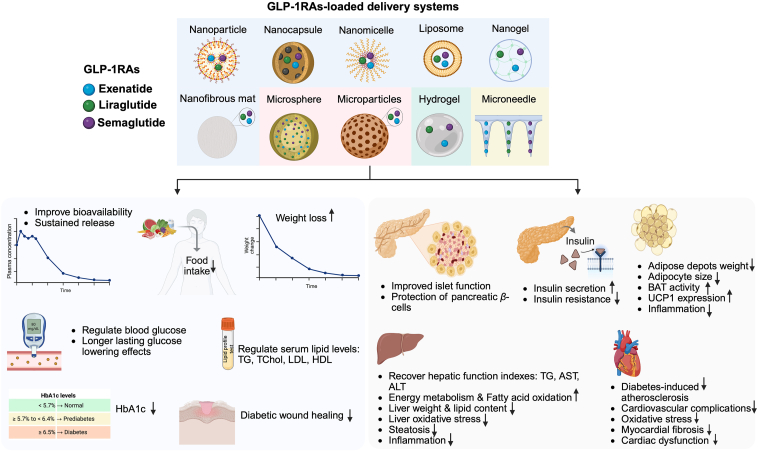
Schematic of advanced drug delivery systems for GLP-1 receptor agonists (GLP-1RAs), illustrating underlying mechanisms of anti-obesity, antidiabetic, and metabolic benefits identified in recent studies.

This review highlights these three drugs (exenatide, liraglutide, and semaglutide), given that most recent studies and innovations have focused on their development and application. Fig. 3 presents a schematic of innovative GLP-1RA delivery systems, highlighting their therapeutic mechanisms responsible for metabolic improvements, glycemic control, and body-weight reduction reported in recent literature. Table 1, Table 2, Table 3, Table 4, Table 5, Table 6, Table 7, Table 8 summarize recent research on the development and application of advanced delivery systems for GLP-1RAs in the management of obesity and diabetes.

**Table 1:** Summary of advancements in NDDSs-based delivery of exenatide for therapeutic intervention in obesity and diabetes.

Therapeutic agent/drug and application	Delivery system type/fabrication method/characteristics	Experimental model	Result	Ref.
Exenatide/diabetes	Polymeric NP/double-emulsification and interfacial cross-linking solidification ▪ Particle size ∼277 nm ▪ Encapsulation efficiency ∼80% ▪ Zeta potential ∼ -–16 (mV) ▪ Polydispersity index ∼0.27 ▪ Dose 30 μg/kg BW ▪ Oral administration	▪ STZ-induced SD diabetic rats	▪ Improved bioavailability ▪ Regulated blood glucose ▪ Regulated serum lipid levels ▪ Improved islet function	192
	Lipid NP/self-emulsifying drug delivery systems containing reverse micelles ▪ Particle size ∼200 nm ▪ Encapsulation efficiency ∼50% ▪ Zeta potential ∼–2.13 (mV) ▪ Polydispersity index <0.2 ▪ Dose 1000 μg/kg BW ▪ Oral administration	▪ Cells: Caco-2, HT-29-MTX, and human Burkitt’s lymphoma cells Raji-B ▪ STZ-induced SD diabetic rats	▪ Enhanced exenatide absorption ▪ Protection of pancreatic *β*-cells ▪ Reduce blood glucose ▪ Reduction in insulin resistance and hyperlipidemia complications	193
	Polymeric NP/ionotropic gelation ▪ Particle size ∼445 nm ▪ Encapsulation efficiency ∼90% ▪ Zeta potential ∼+6.27 (mV) ▪ Polydispersity index ∼ 0.15 ▪ Dose 650 mg/kg BW ▪ Oral administration	▪ Cells: Caco-2, HT-29, and human Burkitt’s lymphoma cells Raji-B ▪ STZ-induced SD diabetic rats	▪ Improved hypoglycemic effect ▪ Enhanced oral absorption	194
	Polymeric NP/– ▪ Particle size ∼156 nm ▪ Encapsulation efficiency ∼52% ▪ Zeta potential ∼+11.3 (mV) ▪ Dose – ▪ Oral administration	▪ *db*/*db* mice	▪ Improved hypoglycemic effects	195
	Polymeric NP/self-assembly and polymer-assisted stabilization ▪ Particle size ∼140 nm ▪ Encapsulation efficiency ∼50% ▪ Zeta potential ∼–22 (mV) ▪ Polydispersity index ∼0.15 ▪ Dose 300 μg/kg BW ▪ Oral administration	▪ Cells: Caco-2, SK-BR-3 ▪ C57BL/6 mice ▪ STZ-induced SD diabetic rats	▪ Enhanced uptake ▪ Increased plasma concentration of Exenatide ▪ Prolonged hypoglycemic effect ▪ Weight loss ▪ Reduced blood lipid levels ▪ Alleviation of organ lesions	196
	Polymeric NP/w/o/w double emulsion ▪ Particle size <200 nm ▪ Encapsulation efficiency ∼60%–70% ▪ Zeta potential ∼–3 (mV) ▪ Polydispersity index ∼0.2 ▪ Dose 0.6 mg/kg BW ▪ Oral administration	▪ *db*/*db* mice	▪ Reduced HbA1C ▪ Long-term hypoglycemic effect ▪ Promoted the % cell repair and proliferation	197
	Polymeric NP/double emulsion ▪ Particle size ∼104–136 nm ▪ Encapsulation efficiency ∼70% ▪ Zeta potential ∼+0.15 (mV) ▪ Polydispersity index ∼0.189 ▪ Dose 100 μg/kg BW ▪ Oral administration	▪ STZ-induced SD diabetic rats ▪ *db*/*db* mice	▪ Greater hypoglycemic response ▪ Improved bioavailability	198
	Polymeric NP/double emulsion ▪ Particle size ∼100 nm ▪ Encapsulation efficiency ∼80% ▪ Zeta potential ∼–4 (mV) ▪ PDI <0.3 ▪ Dose 100 μg/kg BW ▪ Oral administration	▪ Caco-2 cells ▪ *db*/*db* mice	▪ Controlled drug release ▪ Improved bioavailability ▪ Prolonged therapeutic effects ▪ Enhanced drug efficacy ▪ Enhanced insulin secretion and regulate blood glucose levels	199
	Polymeric NP/Hydrothermal ▪ Particle size ∼920 nm ▪ Loading efficiency ∼15% ▪ Zeta potential ∼–8 (mV) ▪ Dose 600 μg/kg BW ▪ Subcutaneous injection	▪ STZ-induced ICR diabetic mice	▪ More controlled reduction of blood glucose ▪ Longer lasting glucose lowering effects	200
	Polymeric NP/Desolvation ▪ Particle size ∼200–300 nm ▪ Encapsulation efficiency ∼50% ▪ Zeta potential ∼–40 (mV) ▪ Polydispersity index ∼0.3 ▪ Dose 100 μg/kg BW ▪ Oral administration	▪ Diabetic *ob*/*ob* mice	▪ Improved bioavailability ▪ Enhanced glucose regulation ▪ Prolonged hypoglycemic effects ▪ Decreased glycated hemoglobin HbA1C ▪ Maintained the body weight	201
	Polymeric NP/spray-drying ▪ Particle size ∼60 nm ▪ Encapsulation efficiency ∼50% ▪ Zeta potential ∼–50 (mV) ▪ Polydispersity index ∼0.14 ▪ Dose 165 μg/kg BW ▪ Oral administration	▪ STZ-induced SD diabetic rats	▪ Improved bioavailability ▪ Enhanced lymphatic uptake ▪ Improved systemic circulation and glucose regulation	202
	Polymeric NP/ionotropic gelation ▪ Particle size ∼156 nm ▪ Encapsulation efficiency ∼50% ▪ Zeta potential ∼7.5 (mV) ▪ Dose 30 μg/kg BW ▪ Oral administration	▪ STZ-induced SD diabetic rats ▪ *db*/*db* mice	▪ Improved bioavailability ▪ Prolong hypoglycemic effects	203
	NP Polymer mixture/layer-by-layer ▪ Particle size ∼167 nm ▪ Loading efficiency ∼3.5% ▪ Dose 10 nmol exenatide/kg BW ▪ Subcutaneous injection	▪ *db*/*db* mice	▪ Reduced blood glucose levels ▪ Enhanced antidiabetic effect over a prolonged period of time	204
	Lipid NP/freeze-drying & layer-by-layer ▪ Particle size ∼154 nm ▪ Encapsulation efficiency ∼80% ▪ Dose 10 nmol exenatide/kg ▪ Subcutaneous injection	▪ *db*/*db* mice ▪ STZ-induced SD diabetic rats	▪ Reduced blood glucose levels ▪ Improved blood glucose control	205
	Nanogel/inverse emulsion polymerization ▪ Particle size <100–200 nm ▪ Loading efficiency ∼14% ▪ Zeta potential ∼–4.6 (mV) ▪ Polydispersity index ∼0.184 ▪ Dose 55 μg/kg BW ▪ Intravenous injection	▪ STZ-induced SD diabetic rats ▪ High-fat diet induced obese C57BL/6 mice	▪ Maintained normoglycemia for ∼48 h ▪ Extended exenatide half-life and provided controlled, biologically timed release ▪ Improved hypoglycemic effects and alleviated diabetes complications ▪ Potential for oral or microneedle administration in future diabetes treatments	206
Exenatide/obesity, NAFLD	Nano-vesicle/dialysis ▪ Particle size ∼253 nm ▪ Encapsulation efficiency ∼64% ▪ Zeta potential ∼+6.48 (mV) ▪ Polydispersity index ∼0.22 ▪ Dose 40 μg/kg BW ▪ Intravenous injection	▪ High-fat diet induced obese C57BL/6 mice	▪ Decreased body weight ▪ Recovered liver lipid content ▪ Recovered hepatic function indexes TG, AST, and ALT	207

Abbreviations: alanine aminotransferase (ALT), aspartate aminotransferase (AST), body weight (BW), glycated hemoglobin A1c (HbA1c), nanocarrier drug delivery systems (NDDS), nanoparticle (np), nonalcoholic fatty liver disease (NAFLD), sprague–dawley (SD), streptozotocin (STZ), triglycerides (TG), Water-In-Oil-In-Water (W/O/W).

**Table 2:** Summary of advancements in microsphere-, hydrogel-, and long-acting formulation-based delivery of exenatide for therapeutic intervention in obesity and diabetes.

Therapeutic agent/drug and application	Delivery system type/fabrication method/characteristics	Experimental model	Result	Ref.
Microsphere-based delivery				
Exenatide/diabetes	Microspheres/– ▪ PLG ▪ Particle size ∼0.06 mm-diameter ▪ Dose 2 mg exenatide encapsulated in 40 mg of microspheres a syringe prefilled with 0.65 mL of diluent ▪ Subcutaneous injection	▪ Human subjects with T2D (Clinical trials phase 2)	▪ Improved glycemic control ▪ Increased reductions in HbA1c and fasting plasma glucose ▪ Decreased gastrointestinal side effects ▪ Extended-release profile ▪ Enhanced flexible dosing ▪ Improved adherence	208
	Microsphere/o/w emulsion solvent evaporation ▪ Particle size ∼20 μ m ▪ Encapsulation efficiency ∼87% ▪ Dose 1 or 2 mg/kg BW ▪ Subcutaneous injection	▪ Zucker diabetic fatty rats ▪ Obese/NAFLD mice	▪ Reduced food intake ▪ Reduced body weight ▪ Reduced HbA1C ▪ Reduced in ALT and AST levels	179,209
Hydrogel-based delivery				
Exenatide/diabetes	Hydrogel/bulk ring-opening copolymerization ▪ PLGA-PEG-PLGA triblock copolymers ▪ Stability >95% at 37 °C over 9 days ▪ Cumulative release >85% over 9 days ▪ Polymer concentration = 25% ▪ Drug loading ∼ 2 mg/mL ▪ Subcutaneous injection	▪ ICR mice	▪ Improved glucose regulation ▪ Suppressed body weight gain ▪ Sustained release of drug	178
Long-acting release formulation				
Exenatide/obesity and diabetes	Long-acting release formulation ▪ Microspheres composed of exenatide and a poly(lactide-*co*-glycolide) polymeric matrix. ▪ Treatments: Placebo or 0.8 or 2.0 mg exenatide Once-weekly subcutaneous injections for 15 weeks ▪ Subcutaneous injection	▪ Randomized, placebo-controlled phase 2 study, subjects with T2D	▪ Reduced body weight ▪ Reduced HbA1C ▪ Improved glycemic control	180

Abbreviations: ALT, alanine aminotransferase; AST, aspartate aminotransferase; BW, body weight; HbA1c, Glycated Hemoglobin A1c; NAFLD, nonalcoholic fatty liver disease; O/W, oil-in-water; PLG, poly(d,l-lactide-*co*-glycolide); PLGA-PEG-PLGA, poly(Lactic-*co*-glycolic acid)-poly(ethylene glycol)-poly(lactic-*co*-glycolic acid); T2D, type 2 diabetes.

**Table 3:** Summary of advancements in MN-based delivery of exenatide for therapeutic intervention in obesity and diabetes.

Therapeutic agent/drug and application	Delivery system type/fabrication method/characteristics	Experimental model	Result	Ref.
Exenatide/diabetes	Dissolving MN/mold-casting ▪ PVA ▪ 145 microneedles ▪ MN height 500 μm ▪ Dose 6.5 μg/mouse	▪ STZ-induced SD	▪ Improved skin permeation ▪ Improved long-term storage stability	210
	Dissolving MN/mold-casting ▪ PVA/PVP/sodium alginate ▪ 225 microneedles ▪ MN height 600 μm ▪ Dose ∼40 μg/mouse	▪ Porcine skin ▪ STZ-induced SD diabetic rats	▪ Sustained plasma concentration over 48 h ▪ Improved bioavailability ▪ Sustained delivery ▪ Sustained hypoglycemic effects	189
	Dissolving MN/mold-casting ▪ Sodium hyaluronate ▪ 225 microneedles ▪ MN height 600 μm ▪ Dose 5 μg/mouse	▪ STZ-induced SD diabetic rats ▪ *db*/*db* mice	▪ Lowered blood glucose levels rapidly ▪ Suppression of food intake	211

Abbreviations: MN, microneedle; PVA, polyvinyl alcohol; PVP, polyvinylpyrrolidone; SD, Sprague–Dawley; STZ, streptozotocin.

**Table 4:** Summary of advancements in NDDSs-based delivery of liraglutide for therapeutic intervention in obesity and diabetes.

Therapeutic agent/drug and application	Delivery system type/fabrication method/characteristics	Experimental model	Result	Ref.
Liraglutide/obesity	Polymeric NP/ion gelation ▪ Particle size ∼253 nm ▪ Encapsulation efficiency ∼72% ▪ Zeta potential ∼+25 (mV) ▪ Polydispersity index ∼0.68 ▪ Dose 0.1–0.4 mg/kg BW ▪ Oral administration	▪ Diet-induced obese ▪ Swiss albino mice	▪ Reduced food intake, body weight, blood glucose, serum total cholesterol, triglyceride, serum resistin, serum insulin level, liver weight, abdominal white adipose tissue, and hepatic oxidative stress	138
Liraglutide/diabetes	Polyphenol-metal (ternary) NP/hydrogen bonding and complex coordination with Al^3+^ ▪ Particle size ∼50 nm ▪ Encapsulation efficiency ∼70%–100% ▪ Zeta potential ∼−20 (mV) ▪ Polydispersity index ∼0.13 PDI ▪ Dose 2–10 mg/kg BW ▪ IP injection	▪ Caco-2 cells ▪ BALB/c mice ▪ *db*/*db* mice	▪ Sustained release ▪ Reduced oxidative stress and cell apoptosis ▪ Improved weight control ▪ Improved organ health (heart, liver, kidney, and pancreas) ▪ Improve glycemic control ▪ Reduce cardiovascular complications	190
	Nano formulation/Nanoprecipitation ▪ Particle size ∼60 nm ▪ Zeta potential ∼+4.9 (mV) ▪ Polydispersity index ∼0.24 ▪ Dose 10 mg/kg BW ▪ Oral administration	▪ STZ-induced SD diabetic rats ▪ *db*/*db* mice	▪ Enhancing patient adherence ▪ Reduced food intake ▪ Enhanced bioavailability ▪ Enhanced glucose lowering effects ▪ Lower body weight	212
	Polymeric NP/anti-solvent precipitation ▪ Particle size ∼238 nm ▪ Encapsulation efficiency ∼41% ▪ Zeta potential ∼+41 (mV) ▪ Polydispersity index ∼0.099 ▪ Dose 200 μg/kg BW ▪ Oral administration	▪ STZ-induced SD diabetic rats	▪ Reduced glucose levels ▪ Controlled glucose levels	213
Liraglutide/diabetes & Obesity	Polymeric NP/coacervation ▪ Particle size ∼37–50 nm ▪ Encapsulation efficiency ∼90% ▪ Zeta potential ∼–25 (mV) ▪ Polydispersity index ∼0.413 ▪ Dose 332 μg/mL (6 μg per animal) ▪ Intravenous injection	▪ C57BL/6 mice ▪ STZ-induced APOE^−/−^ diabetic mice	▪ Inhibited diabetes-induced atherosclerosis ▪ Alleviated the lipid deposition, fibrosis, and endoplasmic reticulum-related oxidative stress in the aorta ▪ Inhibit endoplasmic reticulum stress ▪ Regulated energy metabolism and fatty acid oxidation ▪ Reduced body weight ▪ Reduced blood glucose levels	214
Liraglutide/diabetes	Oligosaccharide NP/Layer-by-layer ▪ Particle size ∼101 nm ▪ Loading efficiency ∼100% ▪ Zeta potential ∼−35 (mV) ▪ Polydispersity index ∼0.24 ▪ Dose 200 μg/kg BW ▪ Subcutaneously injection	▪ Landrace pigs	▪ Improved glycemic effect	215
	Polymeric NP/a double emulsion solvent diffusion ▪ Particle size ∼92 nm ▪ Encapsulation efficiency ∼74% ▪ Polydispersity index ∼0.186 ▪ Dose 0.3 mg/kg BW ▪ Oral administration	▪ STZ-induced SD diabetic rats	▪ Improved gastrointestinal stability ▪ Rapid reduced blood glucose levels ▪ Steady yet long-lasting hypoglycemic effect	216
	Nano micelle/lysine conjugation, followed by ionic complex formation and lyophilization ▪ Particle size ∼75 nm ▪ Drug content ∼101% ▪ Zeta potential ∼+5.44 (mV) ▪ Polydispersity index ∼0.11 ▪ Dose 5, 10, and 20 mg/mL (6 μg per animal) ▪ Oral administration	▪ SD rats ▪ BALB/c mice ▪ C57BL/6J mice ▪ *db*/*db* mice	▪ Reduced blood glucose level ▪ Reduced HOMA-IR ▪ Lowered HbA1c ▪ Improved *β*-cell mass ▪ Reduced epididymal and inguinal white adipose tissue and brown adipose tissue weight ▪ Reduced adipocyte size ▪ Increased BAT activity and *Ucp1* expression ▪ Reduced expression of inflammation-related genes ▪ No liver toxicity ▪ Reduced hepatic cholesterol, triglyceride, LDL/VLDL ▪ Reduced liver lipid accumulation ▪ Improved hypoglycemic effects	217
	Nanocomposite/multi-step solvent-based process ▪ Particle size ∼160 nm ▪ Encapsulation efficiency ∼76% ▪ Zeta potential ∼−8 (mV) ▪ Polydispersity index ∼0.24 ▪ Dose 0.4 mg/kg BW ▪ Oral administration	▪ Caco-2 cells ▪ STZ-induced diabetic C57BL/6J mice	▪ Sustained release ▪ Improved glucose control ▪ Enhanced bioavailability ▪ Increased liraglutide stability	218
	Nanocomposite/electrostatic self-assembly followed by polymer coating. ▪ Particle size <145 nm ▪ Encapsulation efficiency >90% ▪ Zeta potential ∼−30 (mV) ▪ Polydispersity index ∼0.2 ▪ Dose 15 mg/kg BW ▪ Oral administration	▪ Caco-2 cells ▪ STZ-induced SD diabetic rats	▪ Reduced blood-glucose levels ▪ Decreased intake of food and water ▪ Reduced body weight	219
	Nanofibrous mat scaffold/electrospinning ▪ Dose 0.5 to 1 mg per mat	▪ HUVEC Cells ▪ STZ-induced SD diabetic rats	▪ Improved the healing efficiency of diabetic dermal wounds	220

Abbreviations: BW, body weight, BAT Activity, brown adipose tissue activity, HOMA-IR: homeostatic model assessment of insulin resistance, HUVEC: human umbilical vein endothelial Cells, IP: intraperitoneal injection, NP, Nanoparticle, SD, Sprague–Dawley, STZ, treptozotocin, Ucp1, uncoupling protein 1.

**Table 5:** Summary of advancements in microsphere- and enhanced co-formulation-based delivery of liraglutide for therapeutic intervention in obesity and diabetes.

Therapeutic agent/drug and application	Delivery system type/fabrication method/characteristics	Experimental model	Result	Ref.
Microsphere-based delivery				
Liraglutide/diabetes	Microspheres/W1/O/W2 method combined with the premix membrane emulsification technique ▪ Particle size ∼22 μm ▪ Encapsulation efficiency >90% ▪ Drug loading ∼100 mg/mL ▪ Dose: 12 mg/kg BW, a single ▪ Subcutaneous injection	▪ C57BL/6J mice ▪ Diabetic C57BKS-*db* mice ▪ SD rats	▪ Induced hypoglycemic effects ▪ Long-term glycemic control ▪ Reduced HbA1C ▪ Decreased fasting blood glucose ▪ Improved pancreatic function ▪ Reduced hepatic fat content in the liver ▪ Favorable biological safety profile	221
Enhanced co-formulations				
Liraglutide/diabetes	Nano-formulation combined with SGLT-2 inhibitor	▪ Human subjects with T2D (Clinical trials phase 2)	▪ Improved glucose regulation ▪ Improved lipid profile (reduced cholesterol and triglyceride levels)	188
	Novel co-formulations of a basal insulin and liraglutide	▪ Human subjects with T2D (Review of clinical trials phase 2)	▪ Reduced HbA1C ▪ Reduced fasting plasma glucose ▪ Minimized side effects (*e.g.*, weight gain and hypoglycemia)	187

Abbreviations: BW, body weight, HbA1C, hemoglobin A1C, SGLT-2 inhibitor, sodium-glucose cotransporter-2 inhibitor, SD, Sprague–Dawley, W1/O/W2, water-in-oil-in-water.

**Table 6:** Summary of advancements in MN-based delivery of liraglutide for therapeutic intervention in obesity and diabetes.

Therapeutic agent/drug and application	Delivery system type/fabrication method/characteristics	Experimental model	Result	Ref.
Liraglutide/diabetes	Dissolving MN/mold casting ▪ PVA/sucrose ▪ 96 microneedles ▪ MN height 80 μm ▪ Dose 12.5 μg/kg BW	▪ Pig skin ▪ STZ-induced Sprague–SD diabetic rats	▪ Faster absorption of liraglutide than that provided by subcutaneous injection ▪ Increased bioavailability ▪ Similar patterns of anti-hyperglycemic effect in diabetic rats	222
Liraglutide/obesity	Dissolving MN/mold casting ▪ Hyaluronic acid/propylene glycol ▪ 28 microneedles ▪ MN height 150 μm ▪ Dose 0.2 mg/kg BW	▪ Pig skin ▪ Diet-induced obese C57BL/6J mice	▪ Decreased gonadal fat mass and reduced adipocyte size ▪ Ameliorated hepatic steatosis ▪ Enhanced bioavailability and patient compliance ▪ Efficient transdermal drug delivery	137
Liraglutide/diabetes	Dissolving MN/layer-by-layer casting ▪ Sodium hyaluronate ▪ 21 microneedles ▪ MN height 800 μm ▪ Dose 0.2 mg/kg BW	▪ High-fat diet induced obese/diabetic C57BL/6 mice	▪ Controlled reduced blood glucose levels	223
Liraglutide/diabetes & Obesity	NP-loaded dissolving MN/water/oil/water (w/o/w) emulsion solvent evaporation method/mold-casting NP: ▪ Particle size ∼353 nm ▪ Encapsulation efficiency ∼97% ▪ Polydispersity index ∼0.413 ▪ Dose 3 mg MN: ▪ PLGA/PVP/PVA ▪ 132-270 microneedles ▪ MN height 500 &1000 μm ▪ Dose 0.6–1.8 mg	▪ Wistar rat skin	▪ Enhanced mechanical strength of needles ▪ Eliminated the need for subcutaneous injection ▪ Reduce the amount of drug required for treatment	224

Abbreviations: BW, body weight, MN, microneedle, NP, Nanoparticle, PLGA, poly(lactic-*co*-glycolic acid); PVA, polyvinyl alcohol; PVP, polyvinylpyrrolidone; SD, Sprague–Dawley; STZ, streptozotocin; W/O/W, water-in-oil-in-water.

**Table 7:** Summary of advancements in NDDSs-based delivery of semaglutide for therapeutic intervention in obesity and diabetes.

Therapeutic agent/drug and application	Delivery system type/fabrication method/characteristics	Experimental model	Result	Ref.
Semaglutide/metabolic dysfunction	Lipid nano capsules/phase inversion temperature ▪ Particle size ∼188 nm ▪ Encapsulation efficiency ∼90% ▪ Zeta potential ∼−9.6 (mV) ▪ Polydispersity index <0.2 ▪ Dose 500.0 μg/kg BW ▪ Oral administration	▪ C57BL/6J mice	▪ Reduced fasting glucose ▪ Improved insulin sensitivity and glucose homeostasis ▪ Reduced liver inflammation ▪ Improved bioavailability and therapeutic effects	191
Semaglutide/diabetes	Polymeric NP/w/o/w double emulsion technique ▪ Particle size <200 nm ▪ Encapsulation efficiency ∼60%–70% ▪ Zeta potential ∼−3 (mV) ▪ Polydispersity index ∼0.2 ▪ Dose 3 mg/kg BW ▪ Oral administration	▪ Caco-2 cells ▪ Human intestinal organoids (HIOs) ▪ hFcRn transgenic diabetic mice	▪ Improved glucose regulation ▪ Higher insulin pancreatic content and *β*-cell mass recovery ▪ Anti-inflammatory effects ▪ Superior glucose-lowering effect over a 7-day period	225
	Nanocomplex/solution-based synthesis and polymer coating ▪ Particle size <160–300 nm ▪ Encapsulation efficiency >92% ▪ Zeta potential ∼−14–(−23) (mV) ▪ Polydispersity index ∼0.2 ▪ Dose 0.4 mg/kg BW ▪ Oral administration	▪ Caco-2 cells ▪ STZ-induced SD diabetic rats	▪ Improved weight loss ▪ Glycemic control ▪ Reduced total cholesterol and triglyceride ▪ Reduced HbA1C	226
Semaglutide/diabetic cardiomyopathy	PEGylated nanoliposomes/reverse phase evaporation ▪ Particle size ∼108 nm ▪ Encapsulation efficiency ∼89% ▪ Zeta potential ∼−12 (mV) ▪ Polydispersity index ∼0.24 ▪ Dose 10 μg/kg BW ▪ Intravenous injection	▪ STZ-induced SD diabetic rats	▪ Reduced fasting blood glucose and HbA1c ▪ Reduced oxidative stress, myocardial fibrosis, and cardiac dysfunction	227

Abbreviations: BW, body weight; HbA1c, hemoglobin A1c; NP, nanoparticle; SD, Sprague–Dawley; STZ, streptozotocin.

**Table 8:** Summary of advancements in long-acting hydrogel-based delivery of semaglutide for therapeutic intervention in obesity and diabetes.

Therapeutic agent/drug and application	Delivery system type/fabrication method/characteristics	Experimental model	Result	Ref.
Semaglutide/obesity and Diabetes	Long-acting hydrogel-based depot/polymer–nanoparticle hydrogel formation *via* dual-syringe mixing ▪ PEG-PLA, HPMC-C12 polymers ▪ Dose: 500 μL ▪ Subcutaneous injection	▪ Diabetic rat	▪ Regulated blood glucose levels ▪ Enhanced insulin secretion and sensitivity ▪ Reduce appetite ▪ Reduced HbA1C ▪ Improved biocompatibility ▪ Enabled months-long-acting treatments ▪ Serum creatinine levels remained stable or slightly declined in animals receiving	228

Abbreviations: PEG-PLA, poly(ethylene glycol)-*block*-poly(lactic acid); HPMC-C12, hydroxypropyl methylcellulose dodecyl.

### 8.1 Exenatide

#### 8.1.1 Exenatide-loaded NDDSs

Exenatide-loaded NPs have been developed and synthesized using materials such as polyethylene glycol–poly(lactic-*co*-glycolic acid) (PEG-PLGA), hydroxypropyl methylcellulose, chitosan, phospholipids, ursodeoxycholic acid, DOTAP, Maisine, and Labrasol. These materials have enabled various carrier types to optimize exenatide delivery for diabetes and obesity treatment^192^, ^193^, ^194^, ^195^, ^196^, ^197^, ^198^, ^199^, ^200^, ^201^, ^202^, ^203^, ^204^, ^205^. Various fabrication techniques have been employed, including emulsification, spray drying, polymerization, hydrothermal synthesis, layer-by-layer assembly, freeze-drying, and ionotropic gelation. Evaluations conducted across multiple cell lines (*e.g.*, Caco-2, SK-BR-3, HT-29) and animal models (*e.g.*, *ob*/*ob* and *db*/*db* mice, APOE^−^/^−^ mice, diabetic rats, and pigs) using diverse dosing regimens have shown that these formulations offer several advantages. These include controlled drug release, enhanced bioavailability, prolonged therapeutic action, improved glucose regulation, reduced HbA1c, sustained glucose lowering, decreased insulin resistance, better management of body weight, regulated serum lipid profiles, and a reduction in organ damage and related complications. Overall, these formulations show superior antidiabetic and anti-obesity effects over extended periods.

Beyond NPs, innovative nanogels and nanovesicles have further advanced exenatide delivery and therapeutic outcomes. Bai et al.^206^ developed lectin Concanavalin A-modified oxidized starch nanogels for glucose-responsive delivery of exenatide. Tested at 55 μg/kg in Streptozotocin (STZ) and diet-induced obese C57BL/6J mice, these nanogels maintained normoglycemia for approximately 48 h by extending exenatide’s half-life and providing controlled release aligned with biological rhythms. The system effectively improved hypoglycemic effects while alleviating diabetes complications, with researchers suggesting potential applications for oral or microneedle administration in future diabetes treatment strategies. Xie et al.^207^ developed ursodeoxycholic acid-modified oligochitosan nanovesicles for the co-delivery of exenatide and ursodeoxycholic acid using a simple self-assembly method. In high-fat diet-induced obese C57BL/6 mice treated with 40 μg/kg, these exenatide-loaded vesicles selectively targeted the liver, reduced body weight, improved liver lipid content, and normalized hepatic function markers, including triglycerides, aspartate aminotransferase, and alanine aminotransferase. Mechanistically, the vesicles activated sirtuin 1 (*Sirt1*) and suppressed peroxisome proliferator-activated receptor gamma coactivator 1 *β* (*Pgc1b*) and peroxisome proliferator-activated receptor *γ* (*Pparg*), thereby reducing liver fat deposition and restoring liver function. These results demonstrate a promising strategy for the treatment of nonalcoholic fatty liver disease (NAFLD) and obesity. Table 1 192, 193, 194, 195, 196, 197, 198, 199, 200, 201, 202, 203, 204, 205, 206, 207 presents a summary of these studies.

#### 8.1.2 Exenatide-loaded microspheres

DeYoung et al.^208^ developed a once-weekly exenatide (EQW) formulation by encapsulating the drug in biodegradable poly-(d,l-lactide-*co*-glycolide) (PLG) microspheres, with each microsphere containing 5 mg of exenatide per 100 mg of microspheres. The EQW kit includes a pre-measured vial with 2 mg of exenatide in 40 mg of microspheres, along with a syringe, diluent, and connector for easy patient self-administration at any time. After injection, the microspheres gradually release exenatide, reaching steady therapeutic levels in 6–7 weeks. This extended-release profile improves HbA1c and fasting plasma glucose, reduces gastrointestinal side effects, and offers greater dosing flexibility, supporting better adherence and glycemic control in adults with T2D. In another study, Son et al.^179^ investigated a new sustained-release microsphere formulation of exenatide, known as DA-3091. Their study found that monthly or biweekly subcutaneous injections significantly reduced body weight gain in obese mice and improved liver health in mouse models of obesity and NAFLD by lowering serum liver enzymes and reducing hepatic lipid accumulation. Similarly, Kwak et al.^209^ tested DA-3091 in a rat model of T2D and found that administering the treatment every three weeks led to dose-dependent reductions in fasting blood glucose levels, HbA1c, food intake, and body weight. The efficacy of DA-3091 was comparable to that of traditional exenatide injections given twice daily. Together, these studies suggest that DA-3091 is a promising therapeutic candidate for obesity, NAFLD, and T2D, as it offers effective metabolic control with less frequent dosing, potentially improving patient compliance. Table 2 178, 179, 180^,^^208^^,^^209^ presents a summary of these studies.

#### 8.1.3 Exenatide-loaded hydrogel

Li et al.^178^ developed a long-acting injectable exenatide formulation for T2D using a thermosensitive PLGA-PEG-PLGA hydrogel. By adding zinc acetate, PEG, and sucrose, they minimized the initial burst release and achieved steady, sustained exenatide delivery over one week. In mice, a single injection maintained stable blood glucose, improved glucose tolerance, and reduced weight, highlighting the promise of this hydrogel system for sustained peptide delivery in diabetes treatment. Table 2 presents a summary of this study.

#### 8.1.4 Exenatide-loaded MNs

In addition, MN delivery systems have demonstrated significant potential. Studies by Liu et al. and Zhu et al.^189^^,^^210^^,^^211^ have developed and fabricated polymeric, dissolving exenatide-loaded MNs with heights ranging from 500 to 600 μm and patch arrays containing 145 to 225 microneedles, delivering 5–40 μg per mouse using a mold-casting method. These systems have shown enhanced skin permeation, improved long-term storage stability, sustained plasma concentrations for up to 48 h, increased bioavailability, rapid reduction of blood glucose levels, and suppression of food intake. Furthermore, they have demonstrated extended hypoglycemic effects in diabetic rats and *db*/*db* mice. Table 3 ^189^^,^^210^^,^^211^ presents a summary of these studies.

#### 8.1.5 Long-acting exenatide formulation

A phase 2 randomized, placebo-controlled study found that once-weekly long-acting exenatide significantly improved glycemic control and promoted weight loss in patients with inadequately controlled T2D. After 15 weeks, long-acting exenatide led to dose-dependent reductions in HbA1c, fasting glucose, and postprandial hyperglycemia, with 86% of high-dose patients reaching A1C ≤ 7%. The higher dose also resulted in a weight loss of 3.8 kg. Both doses were well tolerated, with mild nausea as the most common side effect^180^. Table 2 presents a summary of this study.

### 8.2 Liraglutide

#### 8.2.1 Liraglutide-loaded NDDSs

Advanced nanocarrier systems have been designed to enhance the delivery of liraglutide for the treatment of obesity and diabetes. Various NP-loaded liraglutide formulations have been developed^138^^,^^190^^,^^212^, ^213^, ^214^, ^215^, ^216^ using materials such as polyphenol–metal complexes, oligosaccharides, zein, chitosan, poly(ethyl acrylate, methyl methacrylate, and chlorotrimethylammonioethyl methacrylate), and PLGA. These formulations are prepared using techniques such as the layer-by-layer method, nanoprecipitation, anti-solvent precipitation, double emulsion, coacervation, ion-gelation, lysine conjugation with lyophilization, and jet mixing. Their effectiveness has been demonstrated in multiple animal models, including diabetic and obese mice and rats. NP-based liraglutide delivery systems have demonstrated improved patient adherence, enhanced bioavailability, and sustained glucose-lowering effects. These formulations also reduce body weight, food intake, cardiomyopathy, and lipotoxicity, while improving heart health by lowering triglyceride, diacylglycerol, and protein kinase C levels and reducing oxidative stress and cell death. Additional benefits include better lipid profiles, lower insulin and resistin levels, decreased liver and adipose tissue mass, and reduced hepatic oxidative stress. Notably, they help prevent diabetes-related atherosclerosis, minimize lipid accumulation and fibrosis in the aorta, and support healthy energy metabolism. Improved gastrointestinal stability and rapid, long-lasting hypoglycemic action further highlight the potential of NP-based liraglutide systems for managing diabetes and obesity.

In addition to NP systems, other nanocarrier systems such as nanomicelles, nanocomposites, and nanofibrous mats have been developed and studied for liraglutide delivery. Subedi et al.^217^ developed a liraglutide-loaded methyl-*β*-cyclodextrin-based nanomicelle using lysine conjugation, followed by ionic complex formation and lyophilization. Treatment with this formulation in *db*/*db* mice resulted in significant antidiabetic and anti-obesity effects, including reduced blood glucose levels, Homeostatic Model Assessment of Insulin Resistance, and HbA1c; improved *β*-cell mass; decreased weights of epididymal, inguinal, and brown adipose tissues; reduced adipocyte size; increased brown adipose tissue activity and uncoupling protein 1 (*Ucp1*) expression; lower expression of inflammation-related genes; no liver toxicity; reduced hepatic cholesterol, triglycerides, LDL/VLDL, and liver lipid accumulation; and improved hypoglycemic effects. Furthermore, Bao et al.^218^ and Song et al.^219^ have created liraglutide-loaded polymeric nanocomposites using zein biopolymers and poly(methacrylic acid-*co*-methyl methacrylate), respectively, *via* multistep solvent-based processes and electrostatic self-assembly, followed by polymer coating methods. *In vitro* and *in vivo* studies using Caco-2 cells, diabetic C57BL/6J mice, and diabetic rat models showed promising results, including sustained drug release, increased liraglutide bioavailability and stability, reduced blood glucose levels, decreased food and water intake, and reduced body weight. Additionally, Yu et al.^220^ fabricated PLGA/gelatin electrospun nanofibrous mats, which improved the healing efficiency of diabetic dermal wounds in diabetic rats. Table 4 ^138^^,^^190^^,^^212^, ^213^, ^214^, ^215^, ^216^, ^217^, ^218^, ^219^, ^220^ presents a summary of these studies.

#### 8.2.2 Liraglutide-loaded microspheres

Gao et al.^221^ developed liraglutide-loaded microspheres, achieving a sustained release of liraglutide over one month. In *db*/*db* mice, a single subcutaneous injection of the microspheres at a dose of 12 mg/kg was compared to daily liraglutide injections (0.2 mg/kg, twice daily) over a 32-day treatment period. The microspheres maintained fasting blood glucose and HbA1c at reduced levels for the entire month, with efficacy comparable to daily injections. Additionally, the formulation improved pancreatic and liver function, showed high drug loading (>8%) and encapsulation efficiency (>85%), and exhibited a low initial burst release. These results support the potential of once-monthly liraglutide microspheres to enhance glycemic control and patient compliance in T2D management. Table 5 ^187^^,^^188^^,^^221^ presents a summary of this study.

#### 8.2.3 Liraglutide-loaded MNs

Lin et al.^222^ and You et al.^223^ developed dissolving, liraglutide-loaded MNs using sodium hyaluronate and polyvinyl alcohol/sucrose *via* mold and layer-by-layer casting. In diabetic and obese mouse models, these MNs enabled faster liraglutide absorption, greater bioavailability, and effective glucose control, while also reducing body weight, cardiomyopathy, lipotoxicity, and oxidative stress. In another study, Juhng et al.^137^ fabricated dissolving MN using mold casting with hyaluronic acid and propylene glycol. When tested in diet-induced obese C57BL/6J mice, this system decreased gonadal fat mass, reduced adipocyte size, ameliorated hepatic steatosis, and enhanced both bioavailability and patient compliance, demonstrating efficient transdermal drug delivery. Rabiei et al.^224^ developed dissolving polymeric MN loaded with liraglutide NP, with a loading capacity of 0.6–1.8 mg of drug, and tested them *in vitro* using Wistar rat skin. This combined (MN and NP) approach improved mechanical strength, showed potential to replace subcutaneous injections, and reduced the required drug dose, highlighting significant advantages for obesity and diabetes therapy. Table 6 ^137^^,^^222^, ^223^, ^224^ presents a summary of these studies.

#### 8.2.4 Enhanced liraglutide co-formulations

A study conducted by She and Liu^188^ evaluated the effects of combining a liraglutide nano-formulation with an SGLT-2 inhibitor in patients with T2D. The study involved 30 participants who were divided into three treatment groups. Those who received the combination therapy demonstrated significant improvements in their lipid profiles after just three days. Specifically, there were notable reductions in cholesterol and triglyceride levels, along with a 25.6% increase in lipid metabolism. These findings suggest that the combination of liraglutide nano-formulation and SGLT-2 inhibitor may provide effective therapeutic benefits for improving blood lipid regulation in individuals with T2D. In another study, Wysham et al.^187^ reviewed key clinical trials demonstrating that novel fixed-ratio co-formulations of basal insulin analogs and GLP-1RAs, such as IDegLira (insulin degludec/liraglutide) and iGlarLixi (insulin glargine/lixisenatide), provide effective new treatment options for patients with T2D who are not meeting glycemic targets with oral agents or single therapies. These trials showed that, in insulin-naive patients, these combinations achieved greater reductions in A1C and fasting plasma glucose than either component alone, with a high proportion of patients reaching A1C targets below 7%. Additionally, the co-formulations minimized side effects like weight gain and hypoglycemia and offered the convenience of a single daily injection, supporting their use as a practical and safe intensification strategy for poorly controlled T2D. Table 5 presents a summary of these studies.

### 8.3 Semaglutide

#### 8.3.1 Semaglutide-loaded NDDSs

Nanocarrier systems loaded with semaglutide, including NPs, nanoliposomes, and nanocomplexes, have been developed to target diabetes and related metabolic disorders^191^^,^^225^, ^226^, ^227^. They were evaluated in different animal models, including C57BL/6J mice, transgenic diabetic mice, and diabetic rats, at doses from 10 μg/kg to 3 mg/kg body weight. Across this dose range, the formulations provided multiple therapeutic benefits: they significantly reduced fasting glucose and HbA1c levels, improved insulin sensitivity and glucose homeostasis, enhanced glucose regulation, and increased pancreatic insulin content and *β*-cell mass recovery. Additional advantages included decreased liver inflammation, reduced oxidative stress, and notable anti-inflammatory effects. The nanocarrier systems also improved bioavailability, delivered superior glucose-lowering effects over days, and reduced myocardial fibrosis and cardiac dysfunction. These findings emphasize the potential of these formulations to effectively manage diabetic cardiomyopathy and other metabolic disorders. Table 7 ^191^^,^^225^, ^226^, ^227^ presents a summary of these studies.

#### 8.3.2 Semaglutide-loaded hydrogel

d’Aquino et al.^228^ developed an injectable hydrogel depot platform designed for the ultra-sustained release of incretin-based therapies, specifically semaglutide and tirzepatide. In diabetic rat models, a single injection maintained therapeutic drug levels and effectively controlled glycemia and weight for over six weeks. This approach matches the efficacy of daily injections but significantly reduces the dosing frequency. The hydrogel system demonstrated excellent biocompatibility, minimized tissue inflammation, and provided tunable, predictable drug release. These findings emphasize the potential of this hydrogel technology to enhance patient adherence, decrease dosing frequency, and offer safer, long-acting treatment options for chronic metabolic diseases. Table 8 ^228^ presents a summary of this study.

## 9 Summary and future perspective

GLP-1RAs are primarily used to treat obesity and T2D. The mechanisms underlying their functions include stimulating insulin secretion, suppressing glucagon secretion, delaying gastric emptying, and enhancing satiety *via* central nervous system regulation. So far, several GLP-1RAs have been developed and marketed^229^. Table 9 summarizes the key GLP-1RAs, their market status, half-lives, dosing frequencies, and applications. Common side effects include gastrointestinal issues and injection-site reactions. To improve adherence and therapeutic outcomes, novel delivery methods are being explored. Although advanced delivery systems have been developed to overcome the limitations of traditional GLP-1RAs therapies, such as rapid clearance, poor bioavailability, frequent dosing, gastrointestinal side effects, and patient compliance, they also pose some challenges. These include the compatibility of materials and fabrication methods with GLP-1RAs, limitation of drug loading capacity, and the stability and effectiveness of the medication^162^^,^^230^. Additionally, complicated manufacturing processes and technical requirements can limit large-scale production and broad implementation of these delivery systems^22^^,^^116^. Current clinical development of GLP-1RAs delivery systems for T2D demonstrates promising progress. Extended-release injectable formulations, such as once-weekly exenatide encapsulated in PLG microspheres, offer sustained glycemic improvements, modest weight loss, and enhanced dosing flexibility. Liraglutide nano-formulations combined with SGLT-2 inhibitors have shown rapid improvements in lipid profiles, indicating enhanced therapeutic efficacy in small, early-phase studies. Fixed-ratio co-formulations of basal insulin analogs and GLP-1RAs, such as IDegLira and iGlarLixi, have been validated in clinical trials to achieve superior reductions in A1C and fasting plasma glucose compared to single agents, with conveniences like minimized side effects and simplified dosing^180^^,^^187^^,^^188^. While innovative GLP-1RA delivery systems show potential clinical benefits and tolerability, ongoing research with larger, longer-term studies is needed to fully establish their long-term effectiveness, safety, and cost-efficiency before routine clinical use. As these therapies remain in early development, further evidence will be important to ensure their real-world impact and sustainability.

**Table 9:** Summary of marketed GLP-1RAs for therapeutic intervention in obesity and diabetes.

GLP-1RAs	Brand name	Market status	Half-life	Dosing frequency	Approved application	Year 1st marketed	Country of market
Albiglutide	Tanzeum	Withdrawn	∼5–6 days	Once weekly	T2D	2014	USA
Beinaglutide	N/A	Marketed	Within minutes	Three times daily	T2D	2016	China
Dulaglutide	Trulicity	Marketed	∼5 days	Once weekly	T2D	2014	USA, Globally
Exenatide (Extended)	Bydureon/Bydureon BCise	Marketed	Long-acting (weeks/days)	Once weekly	T2D	2012	USA, Globally
Exenatide (Immediate)	Byetta	Marketed	∼2.4 h	Twice daily	T2D	2005	USA, Globally
Liraglutide	Victoza (T2D)/Saxenda (obesity)	Marketed	∼13 h	Once daily	T2D and obesity	2010 (T2D), 2014 (Obesity)	USA, Globally
Lixisenatide	Adlyxin/Lixumia	Marketed	∼3–4 h	Once daily	T2D	2013	USA, Europe
Semaglutide (Injectable)	Ozempic (T2D)/Wegovy (obesity)	Marketed	∼7 days (≈160 h)	Once weekly	T2D and obesity	2017 (Ozempic), 2021 (Wegovy)	USA, Europe, Globally
Semaglutide (Oral)	Rybelsus	Marketed	∼7 days	Once daily	T2D	2019	USA, Europe
Tirzepatide	Mounjaro/Zepbound	Marketed	∼5–7 days	Once weekly	T2D and obesity	2022	USA (FDA)

Abbreviations: GLP-1RAs, glucagon-like peptide-1 receptor agonists, T2D, type 2 diabetes.

## Author contributions

Mehrnaz Abbasi: Conceptualization, Methodology, Investigation, Resources, Writing - Original Draft, Supervision, Funding acquisition. Kai Sun: Writing - Review & Editing. Kevin W. Huggins: Writing - Review & Editing. Braeden Heath: Investigation. Hannah DeLoit: Investigation. Lauren McGinness: Investigation. Kate Mccamy: Investigation.

## Declaration of generative AI in scientific writing

Artificial intelligence (AI) and AI-assisted technologies were not used in writing this manuscript.

## Conflicts of interest

The authors declare no conflicts of interest.
